# Binding Mechanism of Riboswitch to Natural Ligand Elucidated by McMD-Based Dynamic Docking Simulations

**DOI:** 10.1021/acsomega.3c06826

**Published:** 2024-01-10

**Authors:** Gert-Jan Bekker, Yoshifumi Fukunishi, Junichi Higo, Narutoshi Kamiya

**Affiliations:** † Institute for Protein Research, Osaka University, 3-2 Yamadaoka, Suita, Osaka 565-0871, Japan; ‡ Cellular and Molecular Biotechnology Research Institute, National Institute of Advanced Industrial Science and Technology (AIST), 2-3-26, Aomi, Koto-ku, Tokyo 135-0064, Japan; § Graduate School of Information Science, University of Hyogo, 7-1-28 minatojima Minami-machi, Chuo-ku, Kobe, Hyogo 650-0047, Japan

## Abstract

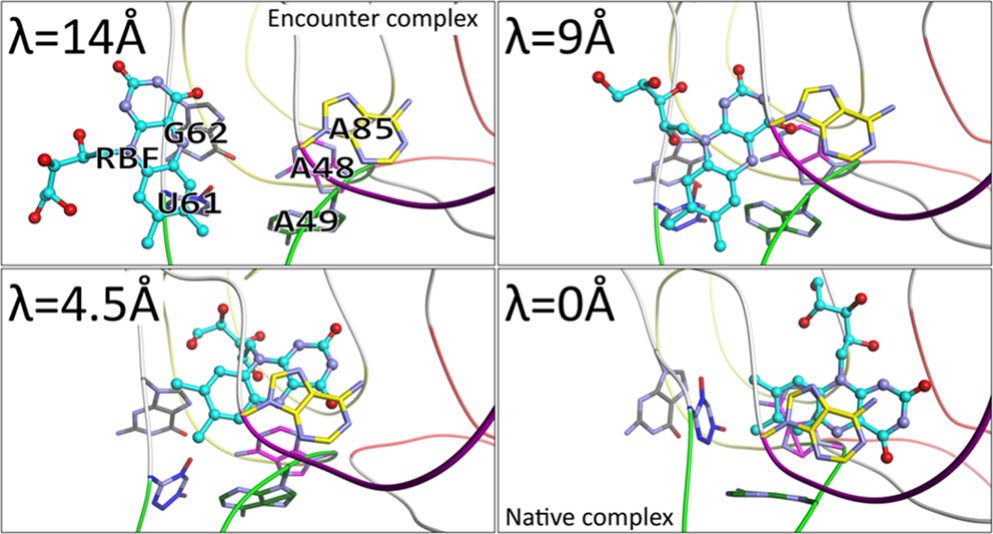

Flavin mononucleotide riboswitches are common among many pathogenic bacteria and are therefore considered to be an attractive target for antibiotics development. The riboswitch binds riboflavin (RBF, also known as vitamin B_2_), and although an experimental structure of their complex has been solved with the ligand bound deep inside the RNA molecule in a seemingly unreachable state, the binding mechanism between these molecules is not yet known. We have therefore used our Multicanonical Molecular Dynamics (McMD)-based dynamic docking protocol to analyze their binding mechanism by simulating the binding process between the riboswitch aptamer domain and the RBF, starting from the apo state of the riboswitch. Here, the refinement stage was crucial to identify the native binding configuration, as several other binding configurations were also found by McMD-based docking simulations. RBF initially binds the interface between P4 and P6 including U61 and G62, which forms a gateway where the ligand lingers until this gateway opens sufficiently to allow the ligand to pass through and slip into the hidden binding site including A48, A49, and A85.

## Introduction

RNA molecules play essential roles in cellular processes^1^ and are hot topics as new therapeutic targets, drug modalities, and vaccines.^2,3^ Noncoding RNAs and untranslated regions (UTRs) regulate gene expression at levels of transcription, RNA processing, and translation, and the RNA structure itself provides biological functions for ribozymes and riboswitches. Noncoding RNAs and UTRs also trigger post-transcriptional events in gene expression, especially in eukaryotes.^4^ Functional UTRs/noncoding RNAs such as riboswitches and rRNAs (rRNAs) have structured elements that form a binding cleft or pocket that small molecules can bind to.^5^ As several natural ligands have been identified, RNA-targeted synthetic drugs that interact with the binding site have also been discovered. For example, an antibiotic, linezolid,^6^ binds to rRNAs to protect from bacterial infection. These compounds could only be found by screening assay experiments because the structural information on RNA is much poorer than that of proteins, reducing the efficiency of rational drug design. In fact, the number of entries in the Protein Data Bank (PDB) are currently roughly 200,000 for proteins, versus roughly 3000 for RNAs.^7^ Although RNAs have properties of more inherently dynamic and less diverse sequence elements than proteins, there is growing interest in developing RNA-targeted small-molecule drugs.^2,8,9^

Riboswitches, which are mainly in the 5′ or rarely in the 3′ UTRs of mRNAs (mRNAs), regulate translation of mRNA by switching its conformation upon small-molecule ligand binding in bacteria.^10^ Riboswitches consist of two modules: an aptamer ligand binding domain and a downstream terminator. Binding of a ligand to the aptamer domain induces a conformational change of the riboswitch, and the subsequent allosteric structural change of the P1 helix enables the formation of the downstream terminator, preventing the binding of a ribosome to the nearby ribosome binding site, halting translation.^11^ On the other hand, as far as we know, mammalian RNAs do not have riboswitches. Therefore, targeting riboswitches with small molecules provides opportunities in the development of new drugs against bacteria.^8^ The flavin mononucleotide (FMN) riboswitch^12^ recognizes riboflavin (RBF, also known as vitamin B_2_) and its metabolic precursors, as well as FMN and flavin adenine dinucleotide (FAD), and complex formation results in transcription termination or translation inhibition, to regulate RBF concentrations.^13,14^ However, RBF and FAD were shown to have relatively low affinity for the riboswitch compared to FMN.^15^ Since the FMN riboswitch is common among many pathogenic bacteria, including Gram-negative bacteria,^14^ it is therefore an attractive target for antibiotics. For example, the FMN riboswitch from *Fusobacterium nucleatum* is associated with periodontitis and was shown to also be prevalent in human colorectal carcinoma.^16^ Experimental structural studies of the aptamer domain, which not only includes both RBF and FMN bound-forms,^17^ but also synthesized ligand bound-forms,^18^ have elucidated their structures, where recently, a high-resolution apo-form was also reported.^19^ To understand how riboswitch domains perform their function, not only structure information but also dynamics information is important. In this aspect, molecular dynamics (MD) simulation studies have contributed to capture the structural and thermodynamic properties of RNAs.^20,21^

Docking using MD simulations,^22^ called dynamic docking, which is one the most powerful computational methods to analyze molecular recognition processes,^23^ can be used to explore binding configurations between receptor proteins and their ligands. We have developed^24,25^ a dynamic docking implementation^26,27^ based on multicanonical molecular dynamics (McMD, see Section S1 for an explanation of the McMD theory),^28^ which we have applied to a number of cases from small-molecule ligands^29,24,25,30^ to medium-sized ligands^31,32^ and peptides.^33−36^ We have also applied McMD simulations to the conformational sampling of proteins and peptides^37,38^ and the loop structure prediction of an antibody,^39^ With McMD, the bias is correlated with the temperature, enabling McMD simulations to adaptively modulate the bias given the density of states. Thus, the potential energy surface functions as a reaction coordinate, which does not depend on any prior knowledge (e.g., native receptor–ligand complex). The canonical ensemble at any given temperature, which is one of the physicochemically acceptable ensembles, can be generated from the multicanonical ensemble by using a reweighting procedure. The free energy landscape (FEL), which governs the thermodynamic properties of a system, can then be obtained by mapping the reweighted structural ensemble onto a reaction coordinate such as a binding path or onto one or more principal components obtained by principal component analysis (PCA).^37,40^ Analysis of the FEL then uncovers the stable bound complexes, as sampled by the McMD simulation. From the stable structures obtained from the subsequent refinement and validation stage using canonical MD simulations, the ligand binding path can be generated by tracing the nearest-neighbor structures from the multicanonical ensemble.

**Figure 1:**
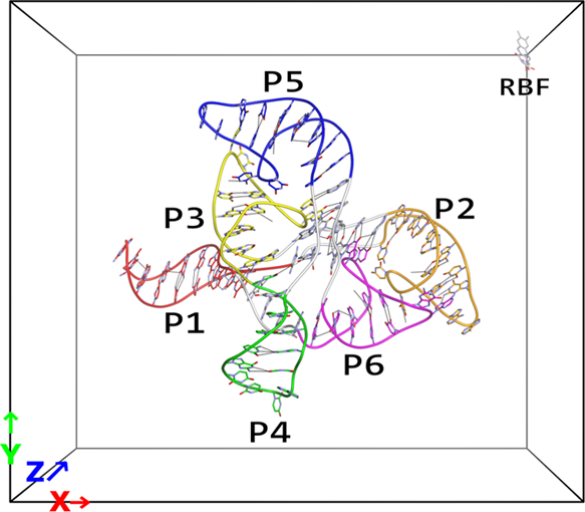
Structural overview of the riboswitch and RBF within the computational system. Shown are the aptamer subdomains P1–P6, colored red (G1-G9, A104-U111), orange (C13-G28), yellow (G33-C46), green (G47-U60), blue (U64-A80), and magenta (A85-G98), respectively, and the natural ligand RBF is shown at the top-right edge of the box. Here, the base sequence, numbering and initial structure correspond to those of the 6WJR structure. Also shown as solid black lines are the distance restraints used during the McMD-based dynamic docking simulations. No restraints were used on the ligand, and it was able to freely sample the space inside the box. The images were produced using Molmil,^74^ a WebGL-based molecular viewer developed by PDBj.^7,56−58^

In order to elucidate the binding mechanism between RBF and the FMN riboswitch, we performed McMD-based dynamic docking simulations between the FMN riboswitch from *F. nucleatum* and its natural ligand RBF (Figure S1), starting from the apo structure of the aptamer, or ligand binding domain, of the riboswitch (Figure 1). The ligand binding domain of the FMN riboswitch consists of six subdomains, identified as P1–P6 helices (simply denoted as P1–P6), forming a duplex structure (with junctions in-between denoted as, e.g., J1–2 for the junction between P1 and P2) that converges in the center, where the binding site is located (Figure 1).^17^ The McMD simulations reproduced the experimental structure along with alternative binding configurations, where the subsequent validation stage of our pipeline identified a binding configuration matching the experimental structure as the most stable one. As the ligand binds deep inside the aptamer in a hidden binding site, binding is modulated by a gateway formed by the backbone of residues J4–5 and J5–6, which can take considerable time to open.

## Results and Discussion

### Wide Configurational Ensemble of the Riboswitch and RBF

**Figure 2:**
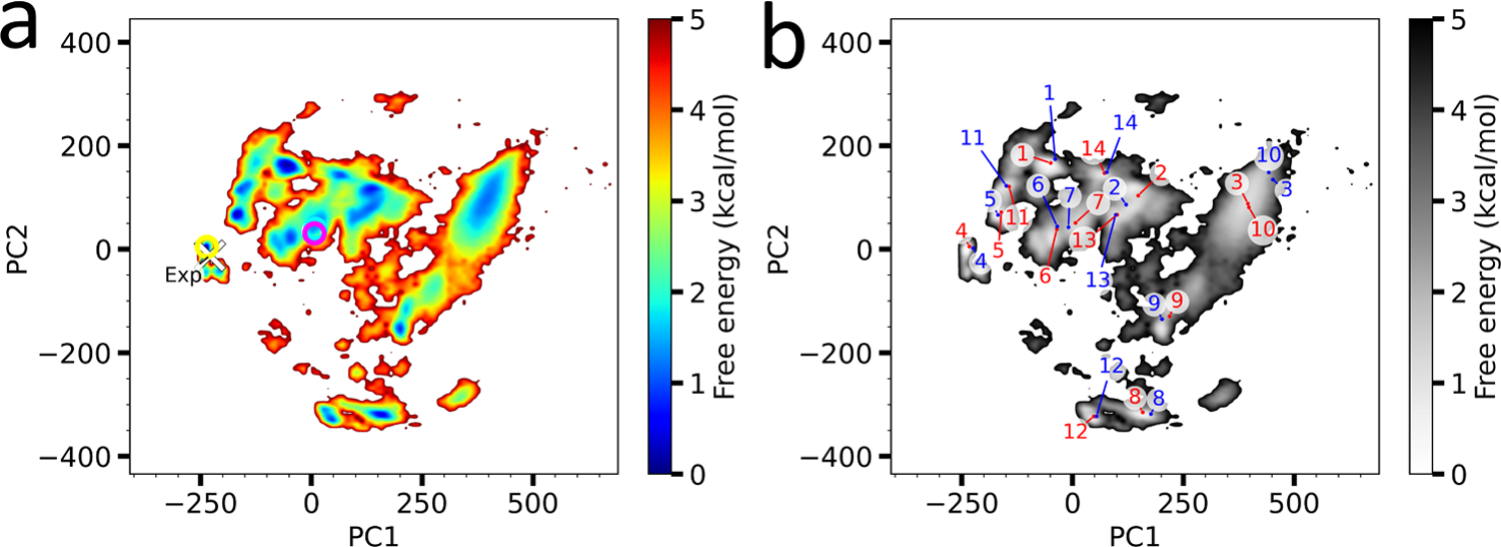
Dynamic docking results between the riboswitch and RBF. (a) Free energy landscape (FEL) of binding configurations along the first and second principal components (PC1 and PC2, respectively), obtained from the reweighted (300 K) multicanonical ensemble. The contour value in kcal/mol is shown on the right side of the figure. The location of the experimental structure (PDB ID 3F4G) is indicated by a white-colored X and indicated with “Exp”. Also shown are the location of the structures shown in Figure 4 as white, cyan, magenta, and yellow circles for (a–d), respectively. (b) FEL with the locations of the representative structures **r**_k_ in red, with the characteristics of these structures listed in Table 1 and the structures themselves shown in Figure S3. In addition, the refined structures **q**_k_ are shown in blue, with the characteristics of these structures listed in Table 2 and the structures themselves shown in Figure S5.

After a 905.24 ns prerun for each parallel McMD trajectory (*N* = 30), the production run was executed, with 2 μs per trajectory (60 μs in total), producing a multicanonical ensemble consisting of 12.0 × 10^6^ structures. Here, we employed distance restraints that restrain the hydrogen bonds formed within the RNA molecule to prevent unfolding at high temperatures during the McMD simulations (Figure 1, see also Section S2). The potential energy distribution obtained from the production run is shown in Figure S2, with the reweighting distributions (eq S5) for *T* at 300 K (room temperature), 500 K, and 700 K. Projecting the ensemble, which was reweighted to room temperature, onto the first two principal axes obtained via PCA (PC1 and PC2), we obtain an FEL (see Section S3) as shown in Figure 2a. The two-dimensional (2D) landscape is very rugged, consisting of a large connected island and multiple smaller ones, with the experimental configuration (PDB ID 3F4G)^17^ located in one of the smaller islands. Within a 1.0 kcal/mol cluster free energy (CFE) cutoff, a relatively large number of representative configurations exist (Table 1). Looking at the location of the representative configurations (Figure 2b), we can see that most of these configurations are located within the large island. One configuration (**r**_4_, the fourth-ranking stable configuration in terms of its free energy at 300 K) is located inside the same basin that contains the experimental configuration, and looking at the characteristics of that configuration (Table 1), we can see that this configuration matches the characteristics of the experimental one. Notably, the ligand’s relative accessible surface area (RASA) of this configuration is considerably lower than that of the other configurations, suggesting that the other configurations are mostly bound on the surface of the riboswitch molecule. Comparing the populations (in terms of their CFEs) between the top four configurations, we can see that they only differ slightly; however, the shape of the islands suggests that the density of **r**_4_ might be higher compared to **r**_1_–**r**_3_ (Figure 2), with **r**_4_ having its own basin, while the other structures share a large basin without distinct borders. Information with respect to the convergence of the pre- and production runs is listed in Tables S1 and S2, respectively.

**Table 1:** Stable Binding Configurations Obtained from the Reweighted (300 K) Multicanonical Ensemble (up to 1 kcal/mol)^a^

	CFE (kcal/mol)	PC1	PC2	RASA	*R*(exp)-value	RMSD (Å)
***r***_1_	0.00	–49.14	166.52	0.43	0.000	17.46
***r***_2_	0.04	148.44	103.71	0.40	0.000	23.60
***r***_3_	0.05	395.73	88.31	0.74	0.000	36.55
***r***_4_	0.10	–232.71	5.09	0.14	1.000	1.63
***r***_5_	0.29	–160.44	71.47	0.34	0.189	10.34
***r***_6_	0.38	–35.24	38.70	0.48	0.018	18.56
***r***_7_	0.39	6.97	50.92	0.59	0.000	20.94
***r***_8_	0.41	158.39	–315.32	0.63	0.000	31.27
***r***_9_	0.62	218.87	–129.48	0.64	0.000	27.21
***r***_10_	0.71	398.08	80.89	0.62	0.000	37.36
***r***_11_	0.71	–143.21	121.21	0.46	0.418	13.09
***r***_12_	0.79	47.91	–323.15	0.44	0.000	25.56
***r***_13_	0.84	100.5	66.64	0.66	0.000	21.79
***r***_14_	0.94	72.00	147.28	0.57	0.000	22.37
exp	-	–228.5	–12.65	0.16	1.000	0.00

a Characteristics for representative structures **r**_k_ (from McMD). Shown are the relative cluster free energy (CFE) value in kcal/mol of the corresponding cluster *k*, the first two principal components (PC1, PC2) coordinates on the FEL in Figure 2, the relative accessible surface area (RASA) of RBF, the *R*(exp)-value, the root-mean-square deviation (RMSD) in Å of the ligand heavy atoms with respect to the experimental structure (PDB ID 3F4G). The *R*-value, which is based on the *Q*-value,^41^ is used to measure the similarity of the interactions,^27,42^ by measuring the change in the contact matrix (between RNA and ligand) for each configuration. The RMSD was calculated over the heavy ligand atoms, after superposing the RNA molecule (backbone phosphate atoms).

**Figure 3:**
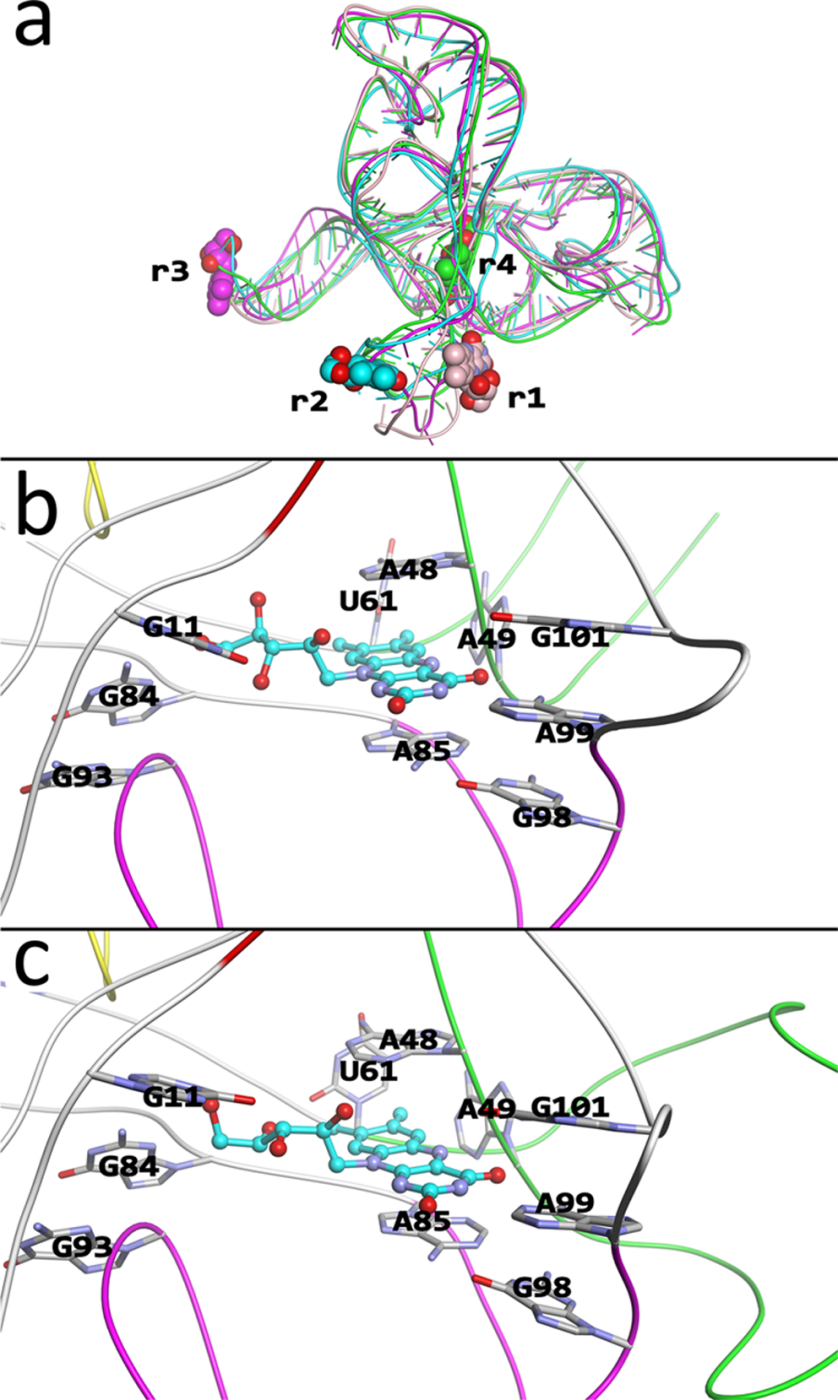
Representative structures identified by the dynamic docking simulations. (a) Overview of the top four ranking structures in pink, cyan, magenta, and green, respectively, with the riboswitch drawn as cartoon, and RBF as space fill. (b) Experimental structure with PDB ID 3F4G and (c) most stable structure **r**_4_ as identified by the validation step in the dynamic-docking pipeline. Shown are the riboswitch as tube model, colored based on the subdomains like in Figure 1, and RBF is shown as ball and stick model in cyan. Also shown and indicated are nearby bases.

Many of the representative configurations have the ligand binding on the surface of the riboswitch molecule (Figures 3a and S3). First, **r**_1_’s ligand binds to P4 near A50 and G51, interacting weakly with the RNA molecule, i.e., no base-stacking, or any specific hydrogen bonding with bases; it simply is positioned on the surface of the RNA molecule, primarily making hydrophobic interactions (perpendicular stacking and ribose interactions). This makes the interaction between the RNA molecule and the ligand quite peculiar in this configuration; the interaction appears to be quite weak, while the ranking from our McMD simulations is quite high. Next, **r**_2_ also binds to P4 in a more interesting manner; it slides directly between A53 and G57, with the bases (U54–U56) looping around the ligand. Even though the configuration is similar to **r**_1_, with **r**_2_ also binding on the surface of the RNA molecule, it, however, binds deeper (even though this is not reflected in the RASA) and makes more specific interactions, both stacking interactions and some hydrogen bonding, albeit with the RNA backbone. Next, **r**_3_’s ligand binds at P1, where the ligand base stacks with G2 and partially with U111 with some hydrogen bonding, stabilizing the termini, but other than that is not making any interesting interactions. We finally get to **r**_4_, which binds deep inside the RNA molecule, in a fashion very similar to the experimental holo structure (Figure 3b,c) with an *R*(exp)-value (see next paragraph) of 1.000 and a root-mean-square deviation (RMSD) of 1.63 Å (Table 1). Here, the ligand base pairs with A99 (P6), while stacking with A85 (P6) and partially with A48 (P4). It also forms hydrogen bonds with G11 (J2–3) and G84 (J5–6), while forming hydrophobic interactions with U61 (J4–5). Unlike the other configurations, this configuration makes more numerous, as well as more stable interactions (i.e., less likely to break due to entropic effects). Next, **r**_5_’s ligand binds at the interface between P4 and P6, and is packed between the backbone of A85 and U59 on the outside of the pocket, and could potentially be an intermediary binding state toward **r**_4_, with the backbone of these bases forming a gateway. The ligand forms stacking interactions with the ribose groups and hydrogen bonds with the phosphate groups, stabilizing the intermediate state. The **r**_6_ (U100), **r**_7_ (U100), **r**_8_ (P3/P5), and **r**_9_ (P1/P3) configurations form stacking interactions and some hydrogen bonds with one of the tail’s OH groups, while **r**_10_ (and to some degree **r**_9_) interacts with the termini by stacking with G1/G2 (P1). Finally, **r**_11_ interacts in a cleft making weak interactions with G98, A49 and C86 (P4/P6), **r**_12_ interacts weakly with C43, U74 and U75 (P3/P5), **r**_13_ interacting weakly with U56 and G57 (P4) and **r**_14_ interacts weakly with G51 and U52 (P4). This is also summarized in Figure S4, which shows the density distribution of the ligand in 3D along the surface of the RNA molecule with high-, medium-, and low-density regions indicated. Notably, **r**_2_, **r**_3_, **r**_7_, **r**_10_, **r**_13_, and **r**_14_ do not occupy high-density regions but only medium- and low-density regions, presumably because these clusters either consist of RNA molecules with a wide conformational variety but a similar binding site (for the higher-ranking clusters) or bind at low affinity (for the lower-ranking clusters).

Our McMD-based dynamic docking simulations have found numerous binding configurations, including several peculiar ones that form seemingly weak interactions but still rank highly. By performing validation simulations (canonical MD simulations at both room temperature as well as high temperature) starting from the structures **r**_k_ without using the distance restraints that were used during the dynamic docking simulations, we can ascertain the relative stability of the binding configurations at 300 and 400 K, as well as obtain refined binding configurations **q**_k_ at 300 K. Table 2 lists some characteristics for the refined structures **q**_k_ (Figure 2b), as well as average *R*-values^31^ and their standard deviations obtained from the simulations. The *R*-value, which is based on the *Q*-value^41^ and can be used to measure the stability of interactions,^27,42,43^ measures the change in the contact matrix (between RNA and ligand) from each initial starting configuration and can be used as a measure for the relative stability of a binding configuration, especially when using high temperature (400 K) MD simulations. The *R*-value ranges from 0 to 1, where higher values (e.g., >0.8) suggest a high conservation of intermolecular interactions. The first thing to note is that **q**_1_, which started from **r**_1_, has an *R*-value at 300 K of only 0.715, suggesting that even at room temperature this structure is quite unstable, which makes it quite peculiar that this structure came as the top-ranking structure from the McMD simulations, as the structure is not particularly stable at room temperature. On the other hand, **r**_4_ is the most stable binding configuration with the highest score at room temperature, as well as at high temperature (and a low standard deviation). Similarly, **r**_2_ is also quite stable at room temperature, as well as at high temperatures, suggesting that **r**_2_ is a potential alternative binding configuration. However, this configuration has a higher standard deviation compared to that of **r**_4_, suggesting that it is more sensitive to entropic effects, potentially due to fluctuations in the RNA molecule at P4, which we also observed in our RBF density analysis (Figure S4). These entropic effects can also be observed when comparing the original structure **r**_2_ with refined structure **q**_2_ (Figure S5), where the structures of P4 differ somewhat, even though the contact matrix should have barely changed, suggesting that P4 underwent some large changes that did not largely affect the interactions. With the other configurations, the ligand mostly binds weakly, with **r**_11_ and **r**_14_ as notable exceptions, although here too, some structural changes are observed when comparing the initial structures with the refined ones.

**Table 2:** Refined Binding Configurations Produced by Canonical MD Simulations and Stability Estimation of r_k_ Using *R*-Value Analysis^a^

	PC1	PC2	RASA	*R*(exp)-value	RMSD (Å)	*R*-value 300 K	*R*-value 400 K
***q***_1_	–38.88	174.07	0.51	0.000	17.58	0.715 (0.284)	0.298 (0.235)
***q***_2_	120.94	86.05	0.39	0.000	22.76	0.999 (0.005)	0.940 (0.113)
***q***_3_	451.97	134.70	0.69	0.000	41.19	0.898 (0.166)	0.490 (0.386)
***q***_4_	–223.90	2.17	0.17	1.000	2.08	0.999 (0.003)	0.990 (0.014)
***q***_5_	–168.02	66.42	0.33	0.190	10.06	0.987 (0.029)	0.394 (0.292)
***q***_6_	–34.49	44.13	0.53	0.008	18.11	0.993 (0.014)	0.223 (0.312)
***q***_7_	–9.07	42.62	0.61	0.000	20.38	0.948 (0.088)	0.385 (0.428)
***q***_8_	177.07	–318.31	0.61	0.000	31.63	0.995 (0.023)	0.416 (0.426)
***q***_9_	202.02	–135.02	0.57	0.000	26.43	0.924 (0.233)	0.084 (0.199)
***q***_10_	442.66	148.35	0.60	0.000	40.83	0.679 (0.301)	0.244 (0.241)
***q***_11_	–149.30	122.56	0.41	0.384	13.22	0.999 (0.002)	0.948 (0.100)
***q***_12_	54.21	–322.58	0.46	0.000	25.34	0.979 (0.080)	0.505 (0.424)
***q***_13_	97.42	66.23	0.66	0.000	21.58	0.854 (0.209)	0.367 (0.319)
***q***_14_	77.73	148.85	0.57	0.001	22.32	0.992 (0.016)	0.976 (0.032)

a Characteristics for representative structures **q**_k_. Shown are the first two principal components (PC1, PC2) coordinates on the FEL in Figure 2, the relative accessible surface area (RASA) of RBF, the *R*(exp)-value, the RMSD in Å of the ligand heavy atoms with respect to the experimental structure (PDB ID 3F4G), the average *R*-value (with respect to the initial structure **r**_k_) of the final 40 ns over 10 parallel trajectories at 300 K (with standard deviation) and the average *R*-value of the final 40 ns over 10 parallel trajectories at 400 K (with standard deviation).

### Ligand Binding Influences the Conformational Ensemble of the Riboswitch

An analysis of the RNA conformation and dynamics might give some insight into how ligand binding might be correlated to a specific RNA conformation. Figure S6 shows the root-mean-square-fluctuation (RMSF) of the reweighted ensemble from our McMD simulations under different conditions; the full ensemble and subensembles that correspond to the clusters of **r**_1_, **r**_2_, **r**_3_, and **r**_4_. Comparing the full ensemble to the individual configuration subensembles, several observations can be made. In the full ensemble, the fluctuation appears to be higher on average compared to those of only **r**_1_–**r**_4_, with a large peak in the P4 helix between helices A50 and U60, which was mostly not included (i.e., not expressed) in the experimental holo structure. Here, **r**_1_–**r**_3_ show lower peaks, while in **r**_4_, the peak is almost nonexistent, suggesting that ligand binding reduces the fluctuation of this highly flexible region, especially when bound to the native binding site. Notably, **r**_3_ binds far away from P4, at the terminal in P1, which showed the highest fluctuation in P4 among the four top-ranking binding configurations. Finally, we have also performed Dynamic Cross Correlation (DCC) of the same subensembles as for the RMSF analysis, which is shown in Figure S7. Here, the full ensemble appears to show a stronger correlation, both positive and negative, compared to the bound structures (especially for **r**_4_, less so for **r**_3_), suggesting that ligand binding also influences the motions and dynamics of the RNA molecule beyond random fluctuations.

Although coordination with Mg^2+^ was observed for other ligands binding to the FMN riboswitch in their crystal structure (e.g., the negatively charged FMN), no coordination of the current RBF ligand was observed primarily because RBF is neutrally charged. While Mg^2+^ is only found at concentrations of 10–30 mM in intracellular environments, most of the Mg^2+^ tends to be bound to ribosomes, polynucleotides, and ATP.^44^ Still, RNA molecules are sensitive to the Mg^2+^ concentration, where an optimal Mg^2+^ concentration leads to a maximum lifetime of an RNA molecule.^45^ To analyze the rate at which Mg^2+^ binds during our McMD simulations, we analyzed the reweighted structural ensemble and found that on average 14.65 Mg^2+^ and 58.77 K^+^ ions were bound simultaneously (i.e., within 5 Å) to the RNA, with the Mg^2+^ binding density shown in Figure S8. During the preparation of the system, 15 Mg and 106 K ions were added to the bulk solvent (randomly), suggesting that RNA is much more likely to bind to Mg^2+^ than to bind to K^+^, and even higher concentrations of Mg^2+^ might also be acceptable. Still, higher Mg^2+^ concentrations might impact the conformational ensemble of the riboswitch or other RNA molecules, and therefore future analysis on the impact of different Mg^2+^ concentrations could help gain insight in how the conformational ensemble is influenced and what concentrations might be optimal when doing MD simulations, considering a limited box size, periodic boundary conditions, and the requirement to have a neutrally charged system.

### Binding Mechanism between RBF and the Riboswitch

**Figure 4:**
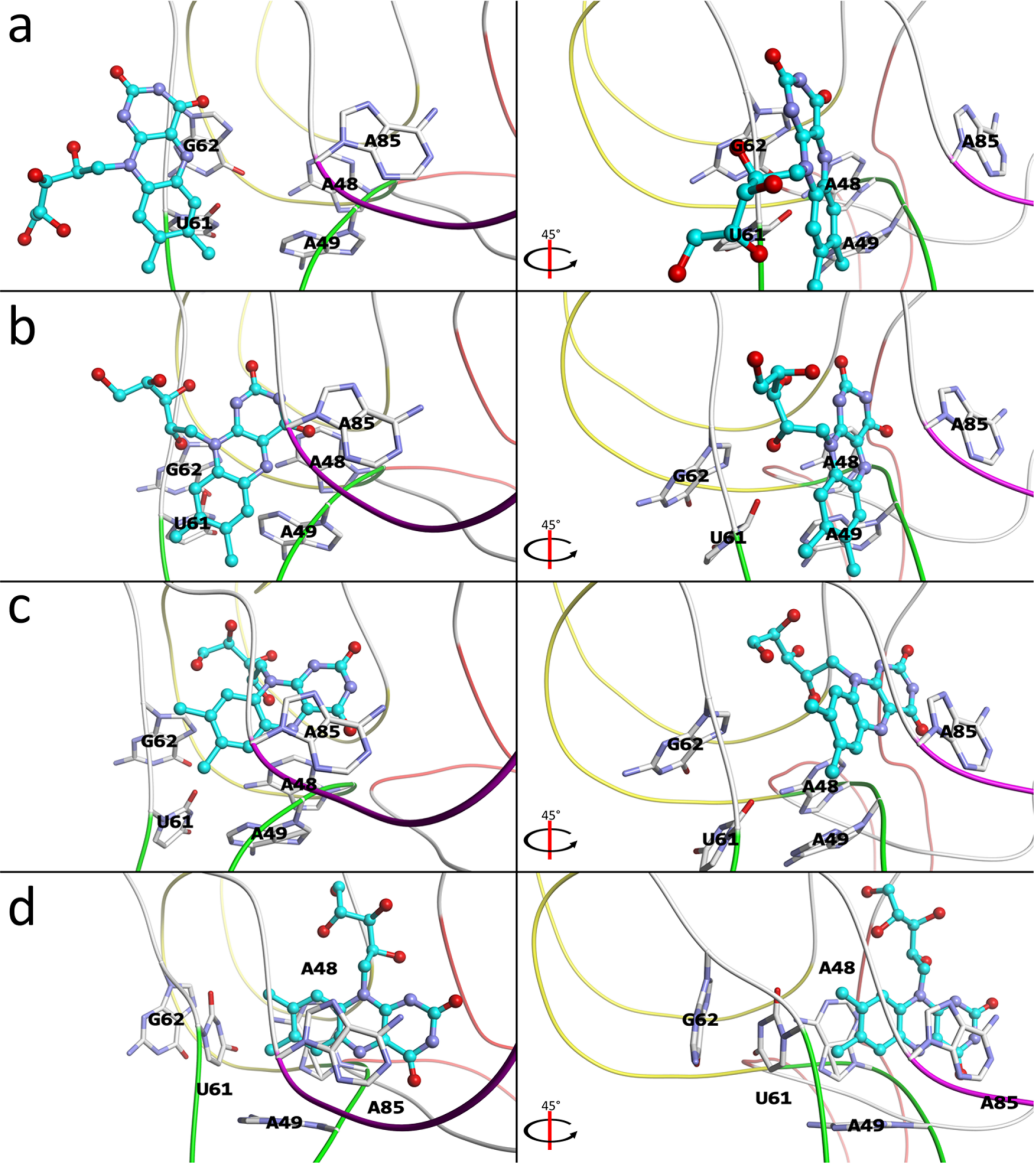
Milestone structures that define the binding mechanism between the riboswitch and RBF. (a) The structure at the window λ = 14 Å. (b) The structure at λ = 9 Å. (c) The structure at λ = 4.5 Å. (d) The structure at λ = 0 Å. In the figure, the riboswitch is shown as a tube model, colored based on the subdomains like in Figure 1, while RBF is shown as a ball and stick model in cyan, with important bases from the riboswitch shown as stick models and indicated. For each subfigure, both a top view with respect to the binding plane (left) and a 45° rotated view (right) are shown. Here, λ is the location along an estimated dissociation vector λ*⇀*, which was determined using our naïve method^24^ starting from the structu**r**e **r**_4_ (d).

By using our McMD-based dynamic docking protocol, not only the experimental binding structure of the FMN riboswitch to RBF has been reproduced (**r**_4_), but also the binding mechanism can be elucidated. Here, to obtain the binding pathway, we used our pathing algorithm^25,27^ starting from the refined structure **q**_4_, where Figure 4 shows several snapshots starting from an outside configuration (Figure 4a), ending in the bound configuration **q**_4_ (Figure 4d), and Figure S9 shows all intermediate structures. Here, the differences between nucleotide conformations are summarized in Table S3, which compares the nucleotides along the binding pathway to **r**_4_ and the experimental structure in terms of their RMSD. Initially, in configuration Figure 4a, the ligand is positioned on top of the ribose groups from U61 and G62 (J4–5), while also forming hydrogen bonds with the phosphate groups of G62 (J4–5) and G84 (J5–6), which together form the gateway into the pocket. In this state, the entrance to the pocket has already been opened somewhat, with the ribose groups of U61 and G62 being pushed further down, thereby opening the entrance to the pocket to some degree. Here, G62 (J4–5) and A48 (P4) form some weak base-pairing, while A49 forms some weak base-pairing with U61 (these do not form base pairs in the **q**_4_ structure). This structure has a gateway size similar to the experimental apo structure, with the distance between A85 and U60 (C4′ – O4′) at 9.65 Å, compared to 9.77 Å for the apo structure.

The structure in Figure 4a looks very similar to that of **r**_5_. However, comparing the structure of **r**_5_ (Figure S3) with the structure shown in Figure 4a, a substantial change can be observed; the structure shown in Figure 4a has the entrance opened more. Here, A49 and U60 form base pairs in Figure 4a, but not in **r**_5_, while the distance between A85 and U61 is 9.6 Å (C4′ – O4′) in Figure 4a, while the distance between A85 – U60 (C4′ – O4′) is 8.5 Å in **r**_5_, where U60 has moved over one base in Figure 4a toward P4, changing the alignment. Therefore, **r**_5_ is the initial encounter complex formed at the interface of P4 and P6, waiting until the P4 conformation has changed, allowing A49 and U60 to base pair and open the gateway for the ligand to then enter into the hidden pocket.

As the ligand moves toward the pocket (Figure 4b), the backbone of U61 and G62 is pushed down further to make more room for the ligand to pass through. The ligand makes many displaced stacking interactions with the surrounding bases (A48, A49, A50), as well as surrounding ribose groups (A49, A85). However, it is mostly stabilized by hydrogen bonding between the ligand’s tail and the phosphate group of G84. The ligand has also initiated a turn, as it is required to turn a total of approximately 90° from the initial configuration (Figure 4a) to the final one (Figure 4d). As the ligand now approaches the binding site (Figure 4c), it has almost completed the 90° turn, with it primarily interacting with A85, and the ligand’s tail with the ribose of C83. Finally, it ends up in the native configuration (Figure 4d), where it stacks between A48 and A85, while base-pairing with A99. It also has perpendicular stacking interactions with A49 and U61. This structure has a gateway width of a smaller size, somewhat similar to the experimental holo structure, with the distance between A85 and U60 (C4′ and O4′) at 3.42 Å, compared to 5.85 Å for the holo structure.

Due to the nature of the gateway (interface of P4 and P6), the orthosteric pocket is a hidden binding site because the gateway effectively hides the presence of the pocket from the ligand. In some way, it could also be considered a cryptic binding site. However, unlike the cryptic binding site of, e.g., the protein Bcl-xL, which is formed due to induced fit with the ligand,^32,35^ the binding site of the riboswitch follows the conformational selection principle. This requires that the conformation of the gateway opens, which based on our simulations seems to be a rare occurrence, as the native configuration was not yet observed early on in our production McMD simulations (Table S2). On the other hand, RBF is unlikely to dissociate from the orthosteric pocket, once bound, partially because the gateway closes again. Experimentally, binding of RBF to the riboswitch was shown to be very weak (*K*_d_ = 39.8 μM).^17^ However, considering what we know about the stability of the molecules in the bound configuration and the behavior of the gateway, the binding affinity between RBF and the riboswitch would be very dependent on the binding constant *k*_on_. Therefore, one would expect the time scale of binding to be very long, potentially so long that most of the complexes formed during the experiment would be non-native, i.e., the **r**_1_–**r**_3_ configurations that we found. While the affinity experiments were only performed over a time span of 10–60 min,^17^ the crystals were formed over a time span of about a week,^17^ giving much more time for the native complex to form and reach an equilibrium. Therefore, binding of RBF to the riboswitch in the hidden binding site is rare, and most of the binding configurations would consist of **r**_1_–**r**_3_, unless enough time is given to reach an equilibrium, both in vitro, as well as in silico.

### Challenges Still Remain to Accurately Model Dynamics of Nucleic Acids

The low ranking of the experimental structure could also be partially attributed to the quality of the force field, to which McMD is quite sensitive, as the enhanced sampling enables the simulation to quickly overcome local minima, providing access to both lower-probability and potentially non-native states. Accurate representation of the RNA is required to be able to accurately sample the conformational space of the RNA molecule during McMD, which influences the configurational space sampled by the RNA–ligand complexes. Previous work has shown that using this force field, the effects of base-stacking are overestimated,^46^ which we also observed, while the effects of base-pairing are underestimated,^47^ which we partially circumvented in our RNA molecule by employing distance restraints to maintain these paired hydrogen bonds. However, we also observed that the backbone is quite rigid and tends to pack quite densely, preventing the gateway from opening easily, while the stacking caused the ligand, which also consists of several ring groups, to become trapped at various locations, including near the RNA backbone. Even though the OL3 force field is a state-of-the-art RNA-based force field,^48^ it may still lack a proper balance between hydrogen bond formation and hydrophobic interactions, compared to modern protein-specific force fields such as AMBER99SB-ILDN,^49^ which we have used extensively in our previous works when docking ligands^26,27^ with the GAFF/GAFF2 force field.^50^ Even complicated docking studies between proteins and ligands, such as the binding of compounds or peptides to cryptic binding sites^51,52^ could successfully be predicted using McMD-based dynamic docking.^32^ Potentially, a modification of the OL3 force field that includes backbone phosphate modifications,^53^ might help in modeling the RNA backbone conformation better. Still, protein force field development has received much greater attention, while nucleotide force fields have lagged behind. Therefore, there is a need for additional nucleotide force field development, where more varied, and complicated systems should be evaluated during its parametrization, as many new RNA structures have been discovered over the past decade.^54^ Similar to protein force field development,^49^ ensuring that the force field can reproduce experimental properties such as sugar, backbone conformations, and hydrogen bonding should lead to a more accurate force field.^55^ This force field development would also benefit from actively making use of enhanced sampling methods such as McMD to ensure that the force field can accurately model the dynamics and free energies of various RNA molecules and their conformations, and not just stabilize known structures at their local minima. As RNA molecules have gained renewed interest in the past decade, improved force fields that can accurately model the complicated dynamics of molecules are vital to advance the field into the next era.

## Conclusions

**Figure 5:**
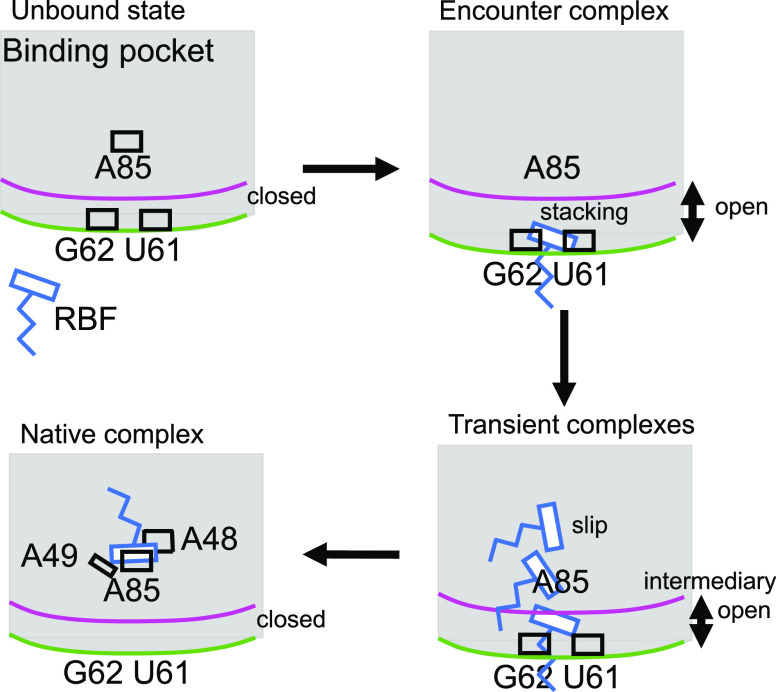
Scheme representing the binding mechanism of RBF to the riboswitch. Shown in a clockwise manner from the top left to bottom left are the distinct phases during binding. Indicated are RBF (blue) some of the bases involved in the binding (black), including the backbone of the riboswitch of J4–5 (green) and J5–6 (magenta).

We have used McMD-based dynamic docking simulations to analyze the binding mechanism between the FMN riboswitch and RBF. We predicted several binding configurations, where the validation and refinement stage in our pipeline correctly identified the experimental structure as the most stable one. Using this structure as a starting point and the structures sampled by our McMD simulation, we established the binding pathway from which we could establish the binding mechanism, which is described schematically in Figure 5. RBF binding is modulated by a gateway formed by the backbone of U61, G62, and G84 at the interface between P4 and P6, where the ligand binds to these backbone nucleotides until the gateway has opened sufficiently for the ligand to pass through and enter the native binding site. We have shown that our dynamic docking protocol can be used to predict binding between an RNA molecule and a small-compound ligand, but that further refinement of RNA force fields (and presumably DNA force fields) is required to more accurately model the interactions between nucleotides and the dynamics of RNA molecules.

## Methods

The apo structure of the *F. nucleatum*’s FMN riboswitch aptamer domain with PDB ID 6WJR (resolution 2.7 Å) was obtained from Protein Data Bank Japan (PDBj),^7,56−58^ where we used the base sequence and numbering that correspond to this structure for the simulations and discussions. We used Gromacs 2021.3^59^ to prepare and perform the simulations, which we modified to perform the dynamic docking and path sampling simulations.^26,27^ In addition, AmberTools and ParmEd were used to generate the RNA parameters and convert them to Gromacs-compatible ones. A box of dimensions 97.4444 × 85.0575 × 61.1150 Å^3^ was placed around the RNA molecule, after which the ligand was placed in one of the corners of the box. Subsequently, the box was solvated, and 0.1 M of KCl was added. In addition, to neutralize the system, 15 Mg^2+^, which corresponds to the number of bound Mg^2+^ observed in the experimental holo structure, and additionally 79 K^+^ were added. The base OL3 force field,^48^ GAFF2,^50^ monovalent^60^ and divalent^61^ ion parameters, and OPC3^62^ were used to parametrize the RNA, the ligand, ions, and water molecules, respectively. For the ligand, Gaussian^63^ at the HF/6-31G* level was used to optimize the geometries and calculate the electron density, followed by RESP^64,65^ to finally obtain the atomic partial charges by fitting the density. The final system consisted of 3572 RNA atoms, 48 ligand atoms, 15,191 water molecules, 15 Mg ions, 106 K ions, and 27 Cl ions. The characteristics of the system are also summarized in Table S4.

NVT simulations were performed at 300 K using the Bussi thermostat,^66^ while the NPT simulations additionally used the Bussi barostat^67^ under 1 bar at 300 K. The long-range electrostatics were calculated using the zero-dipole summation method, which is a cutoff-based approach utilizing a well-defined pairwise function^24,68−70^ with the damping factor α set to 0 Å^–1^ and the atom-based cutoff length set to 12 Å. A time-step of 2 fs was used, with LINCS^71^ to constrain the bond lengths and SETTLE^72^ to constrain the water geometries. Energy minimizations, followed by 100 ps NVT and NPT simulations with position restraints on the heavy solute atoms, were used to prepare the system.

The McMD simulations were executed in a similar manner as our previous works,^27^ with the simulation details described in Section S2. In addition, the dynamic docking analysis and the binding mechanism analyses are described in Sections S3 and S4, respectively.

## Data Availability

The source code for executing McMD dynamic docking simulations, including a modified version of Gromacs and the analysis scripts, are available at https://gitlab.com/gjbekker/gromacs. The representative structures and interactive versions of Figures 1, 3, 4, S3, S4, S5, S8, and S9 have been submitted to the Biological Structure Model Archive (BSM-Arc),^73^ under BSM-00039 (10.51093/bsm-00039).

## Supporting Information Available

McMD algorithm (Section S1); details of the McMD simulations performed (Section S2); McMD dynamic docking analyses (Section S3); binding mechanism analysis (Section S4); 2D chemical structure of the ligand (Figure S1); multicanonical potential energy distribution (Figure S2); top-ranking structures **r**_k_ (Figure S3); density distribution of the ligand sampled during the McMD simulations (Figure S4); comparison of the top-ranking structures **r**_k_ against the refined structures **q**_k_ (Figure S5); comparison of the differences in fluctuation of the RNA molecule of different binding configurations (Figure S6); comparison of the dynamics of the RNA molecule of different binding configurations (Figure S7); density distribution of Mg^2+^ sampled during the McMD simulations (Figure S8); overview of intermediary configurations along the binding pathway (Figure S9); convergence of the McMD prerun (Table S1); convergence of the McMD production run simulations (Table S2); changes of the riboswitch nucleotide conformation as the ligand binds (Table S3); and MD system and simulation parameters (Table S4) (PDF)
