# Supplementary material for: Binding Mechanism of Riboswitch to Natural Ligand Elucidated by McMD-Based Dynamic Docking Simulations
Source: ACS Omega. 2024 Jan 10;9(3):3412–22. doi: 10.1021/acsomega.3c06826 (PMC10809319; doi:10.1021/acsomega.3c06826)
Supplement: Supplementary file 1 — ao3c06826_si_001.pdf [file ao3c06826_si_001.pdf]

Supplementary Information file for:

Binding Mechanism of Riboswitch to Natural Ligand Elucidated by McMD-based Dynamic Docking Simulations

Gert-Jan Bekker[†,*], Yoshifumi Fukunishi[‡], Junichi Higo[§], and Narutoshi Kamiya[§,*]

[†] Institute for Protein Research, Osaka University, 3-2 Yamadaoka, Suita, Osaka 565-0871, Japan.

[‡] Cellular and Molecular Biotechnology Research Institute, National Institute of Advanced Industrial Science and Technology (AIST), 2-3-26, Aomi, Koto-ku, Tokyo 135-0064, Japan.

[§] Graduate School of Information Science, University of Hyogo, 7-1-28 Minatojima Minami-machi, Chuo-ku, Kobe, Hyogo 650-0047, Japan.

Table of contents

**Section S1**	Multicanonical MD algorithm	S3
**Section S2**	McMD-based dynamic docking simulations	S3
**Section S3**	McMD-based dynamic docking analysis	S4
**Section S4**	Binding mechanism analysis	S5
**Fig. S1**	2D structure of the ligand riboflavin	S6
**Fig. S2**	Multicanonical potential energy distribution of the riboswitch – riboflavin dynamic docking	S7
**Fig. S3**	3D structure of picked representative configurations $\boldsymbol{r}_k$ and the experimental structure	S8
**Fig. S4**	Density distribution of RBF in 3D around the RNA	S10
**Fig. S5**	3D structure of picked representative configurations $\boldsymbol{r}_k$ and $\boldsymbol{q}_k$	S11
**Fig. S6**	RMSF analysis of the RNA	S13
**Fig. S7**	Dynamic Cross Correlation analysis of the RNA	S14
**Fig. S8**	Density distribution of Mg^{2+} in 3D around the RNA	S15
**Fig. S9**	Overview of binding pathway structures obtained from the multicanonical ensemble	S17
**Table S1**	Convergence of McMD dynamic docking pre-run simulations	S18
**Table S2**	McMD-based dynamic docking results using subsets of the simulation data	S19
**Table S3**	RMSDs of riboswitch's residues during binding of the ligand RBF along λ	S21
**Table S4**	System & simulation parameters for Riboswitch – RBF binding simulations.	S23
**Supplementary References**		S24

Section S1: Multicanonical MD algorithm

We used our own developed McMD-based dynamic docking method that has been thoroughly described in various previous papers,[1–9] but here we will shortly review the algorithm. The probability distribution of the potential energy of the multicanonical ensemble is defined by the following equation:

$$P_{mc}(E,T_0) = \frac{1}{Z_{mc}} n(E)e^{-W(E)} = constant \tag{S1}$$

where E is the potential energy, T_0 the simulation temperature, $n(E)$ the density of states and Z_{mc} the partition function:

$$Z_{mc} = \int n(E)e^{-W(E)}dE \tag{S2}$$

$W(E)$ is a weighting function to modulate the probability distribution P_{mc} in order for it to become constant and enables the system to take a random walk along the target energy range, and is defined as follows:

$$W(E) = \ln n(E) = \frac{E}{RT_0} + \ln P_c(E,T_0) \tag{S3}$$

where R is the gas constant and P_c the canonical energy distribution at T_0. During the McMD simulations, this weighting function is used to scale the forces by a factor of $\nabla W(E)$, where the multicanonical temperature T_{mc}, which corresponds to $T_0/\nabla W(E)$, is restricted to a specific target range between T_{low} to T_{high}, which we generally set at 280 K and 700 K, respectively. Multiple iterations of sampling are required to estimate the correct bias that enables a random walk along a wide energy range, where the weighting function is updated between iterations using:

$$W^{i+1}(E) = W^i(E) + \ln P^i_{mc}(E,T_0) \tag{S4}$$

After obtaining a flat potential energy distribution, a production run is executed to sample phase space. Due to the bias applied during the McMD simulations, the resulting multicanonical ensemble must be reweighted to obtain the canonical distribution at room temperature. A multicanonical distribution can be reweighted to a canonical distribution at any given temperature T within the flat energy range using the following equation:

$$P_c(E,T) = \frac{1}{Z_c} n(E)e^{-\frac{E}{RT}} = \frac{Z_{mc}}{Z_c} P_{mc}(E)e^{W(E)-\frac{E}{RT}} \tag{S5}$$

Section S2: McMD-based dynamic docking simulations

To prevent unfolding at high temperatures during the McMD simulations and the initial high temperature dissociation simulation, we employed distance restraints that restrain the hydrogen bonds formed within the RNA (Fig. 1), primarily between the base pairs, using a force constant of 1 kcal/mol/Å/Å and a flat bottom distance of 4.5 Å, except for the hydrogen bonds in the terminal bases (at P1), where a force constant of 10 kcal/mol/Å/Å was used.

We used the McMD algorithm described in Section S1, with 30 parallel trajectories initialized with different random seeds for the initial velocity from the initial structure constructed as described in section 1 of the Methods. After initialization, first a 2 ns simulation at T_{high} (= 700 K) was performed for each parallel trajectory to randomize the ensemble with the above-described

weak distance restraints in place. From here, the initial bias was estimated using Eq. S3 and subsequent iterations of increasing simulation lengths were executed, updating the bias using Eq. S4 between iterations, until a sufficiently flat potential energy distribution had been obtained corresponding to a wide T_{mc} range of $T_{low} - T_{high}$, where T_{low} = 280 K, followed by several more iterations for equilibration (Table S1). In total, the pre-run lasted for 27.16 μs (905.24 ns per trajectory). Finally, a 60 μs (2 μs per trajectory) production run was executed to sample the structures including bound and unbound states, which were saved at 5 ps intervals, producing 12.0 x 10^6 structures, with the potential energy distribution shown in Fig. S2.

Section S3: McMD-based dynamic docking analysis

We used the same analyses techniques as we did in our recent works.[10] First, we performed PCA on a distance array derived from the structures. The array consists of riboswitch – riboflavin pairs and riboflavin – riboflavin pairs. From the riboswitch, the backbone phosphate atom, as well as a representative atom from each base was taken (G/A: N1, U/C: N3), while from the ligand the atoms as indicated in Fig. S1 were taken. The distance based approach does not require prior superposition of the structures unlike the quasi-harmonic approach,[4,11] while taking periodic boundary conditions into account and being a more sensitive approach to detecting intermolecular contacts along the entire surface between the interacting molecules. The structures are then projected onto the first two principal components, and the probability of each bin *i* on the landscape is calculated as $P_i = \sum_j P_c(E_j, 300\ K)$ using each structure j within bin i. The free energy as the Potential of Mean Force (PMF) is finally calculated as $PMF_i = -RTln\ P_i$ for each bin, giving the 2D FEL after normalizing its minimum to zero.

After the PCA, we performed K-means clustering on the PCA coordinates of the structures, using K = 1000 clusters and a selected number of PC coordinates, so that the sum of the contribution to their variance exceeds 90 %, here corresponding to PC1-PC8, with each PC contributing 37.02%, 21.34%, 16.52%, 9.92%, 2.74%, 1.36%, 1.09%, 0.94% to the configurational variance. For each cluster k', one representative structure was selected and the clusters were then ranked based on the relative free energy at 300 K of the clusters (cluster free energy, CFE), which was calculated as $PMF_{k'} = -RTln\ P_{k'}$, where $P_{k'} = \sum_j P_c(E_j, 300\ \text{K})$ using each structure j belonging to cluster k'. Then, using these 1000 representative structures, all-to-all R-value analysis.[12,13] was performed. Here, the R-value is a distance-based approach measuring the intermolecular contacts compared to a reference configuration, and is based on the Q-value,[14,15] which measures the fraction native contacts of proteins. Starting from the most stable cluster k'=1 in order of their free energy contribution, similar representative structures in terms of their R-value (R > 0.7) were grouped together and their clusters merged. After re-calculating the CFE of the new clusters, we used a CFE cutoff value of 2.5 kcal/mol to distinguish between potentially interesting structures and less stable ones. This gives us k clusters and their corresponding representative structures $\mathbf{r}_k$.

Representative complex structures $\mathbf{r}_k$ within the CFE cutoff obtained from the multicanonical ensemble were further refined using canonical (NVT) MD simulations at 300 K. For each representative structure, ten 100-ns MD simulations at 300 K were performed (with different random seeds for the initial velocity), with only restraints on the COM of the riboswitch present to prevent translation and rotation of the molecule. Then, refined complex structures $\mathbf{q}_k$ were picked by taking the nearest-to-average structure from the final 40 ns of the canonical MD

simulations. In addition, ten 100-ns canonical MD simulations at 400 K were performed to compare the relative stabilities of the binding configurations, like we have done before.[10]

Section S4: Binding mechanism analysis

We used our previously developed pathing method[12] to construct a binding pathway starting from the equilibrated configuration $\mathbf{q}_4$. As we did not use a cylinder to restrain the sampling region of the ligand, we did not have a reaction coordinate to use for the pathing method. Previously,[8] we developed a naïve method to estimate the optimal unbinding direction $\vec{\lambda}$ of a ligand, given its size, the shape of the pocket and the center of the pocket, and applied it in the same way that we had applied it for our previous work docking Bcl-xL with two medium-sized ligands.[16] Then, using $\mathbf{q}_4$ and the obtained reaction coordinate from the naïve method, we used our pathing algorithm to construct the binding/unbinding pathway. In short, the reaction coordinate is split into pre-defined windows along $\vec{\lambda}$. In each window, one representative structure was picked from the multicanonical ensemble that is similar in terms of its R-value to of the picked structure from the previous window, where for the initial ($\lambda = 0$) window, the structure $\mathbf{q}_4$ was used. This produces a smoothly connected pathway of structures along the dissociation direction $\vec{\lambda}$ starting from the structure $\mathbf{q}_4$ to the outside state (Fig. S9).

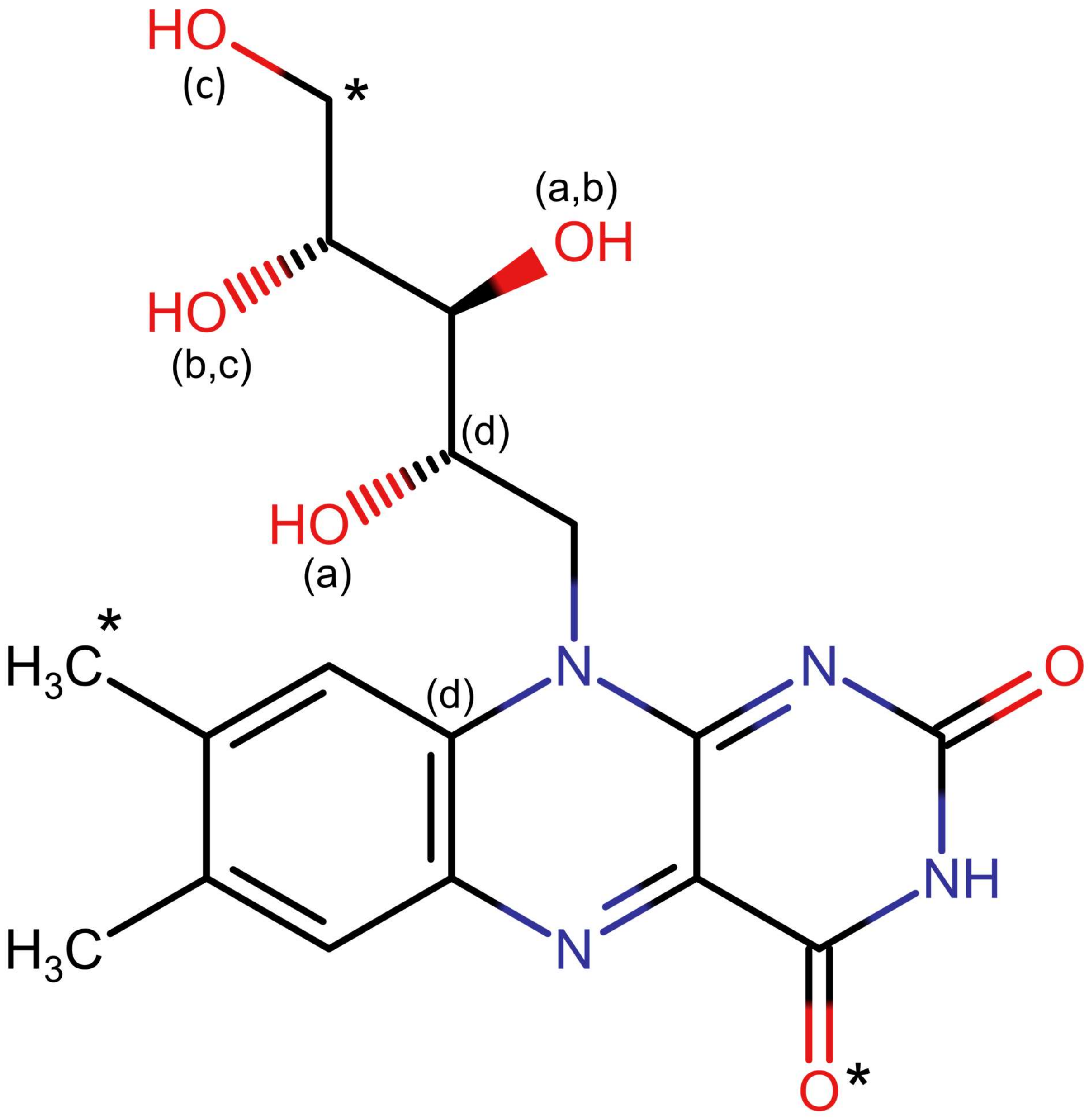

Fig. S1. 2D structure of the ligand riboflavin. Chemical structure of the ligand, with the atoms used to calculate the distance matrix for the PCA indicated. Atoms indicated with a "*" were paired with atoms from the riboswitch, while atoms indicated with a-d were paired with each other.

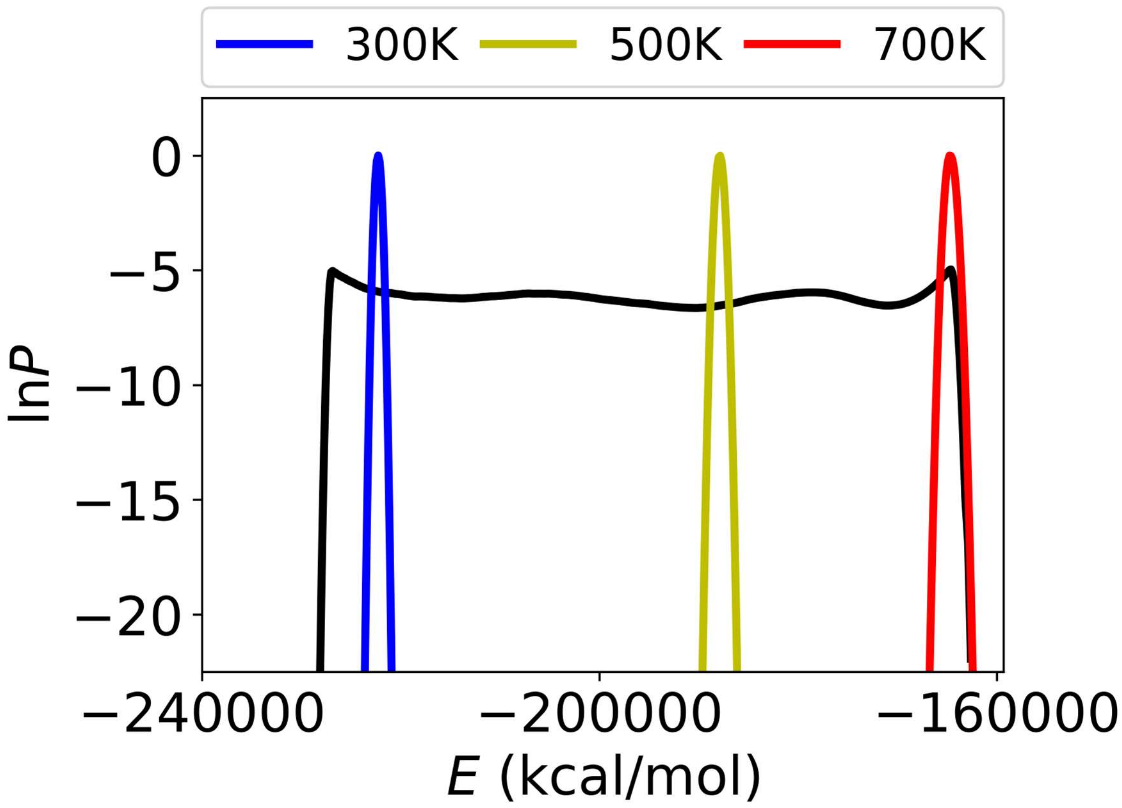

Fig. S2. Multicanonical potential energy distribution of the riboswitch – riboflavin dynamic docking. Potential energy probability distribution ($P_{McMD}(E)$) as sampled during the production run. Also shown are the reweighing canonical distributions ($P_c(E, T)$) at 300 K, 500 K and 700 K in blue, yellow and red, respectively.

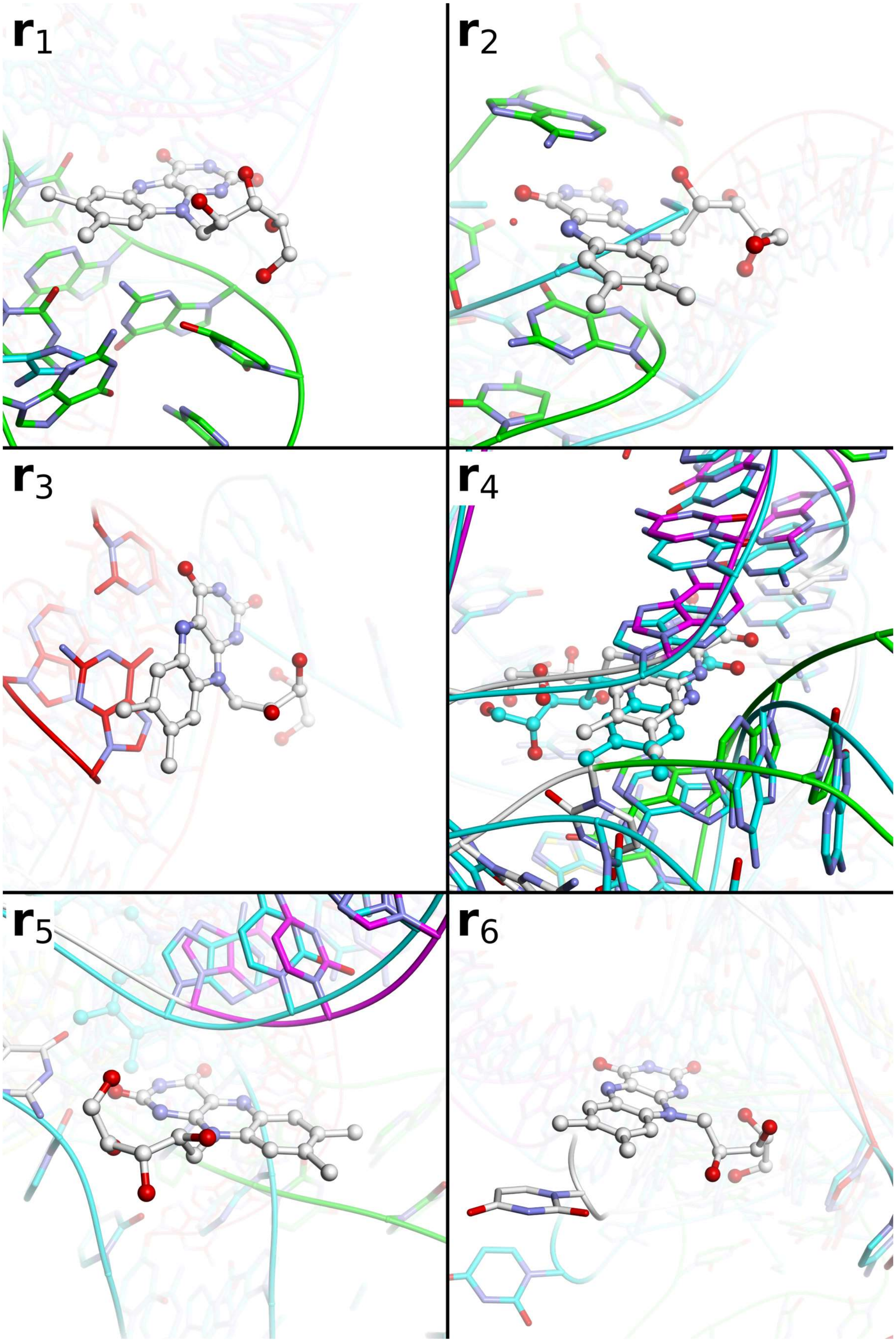
$\mathbf{r}_1$
$\mathbf{r}_2$
$\mathbf{r}_3$
$\mathbf{r}_4$
$\mathbf{r}_5$
$\mathbf{r}_6$

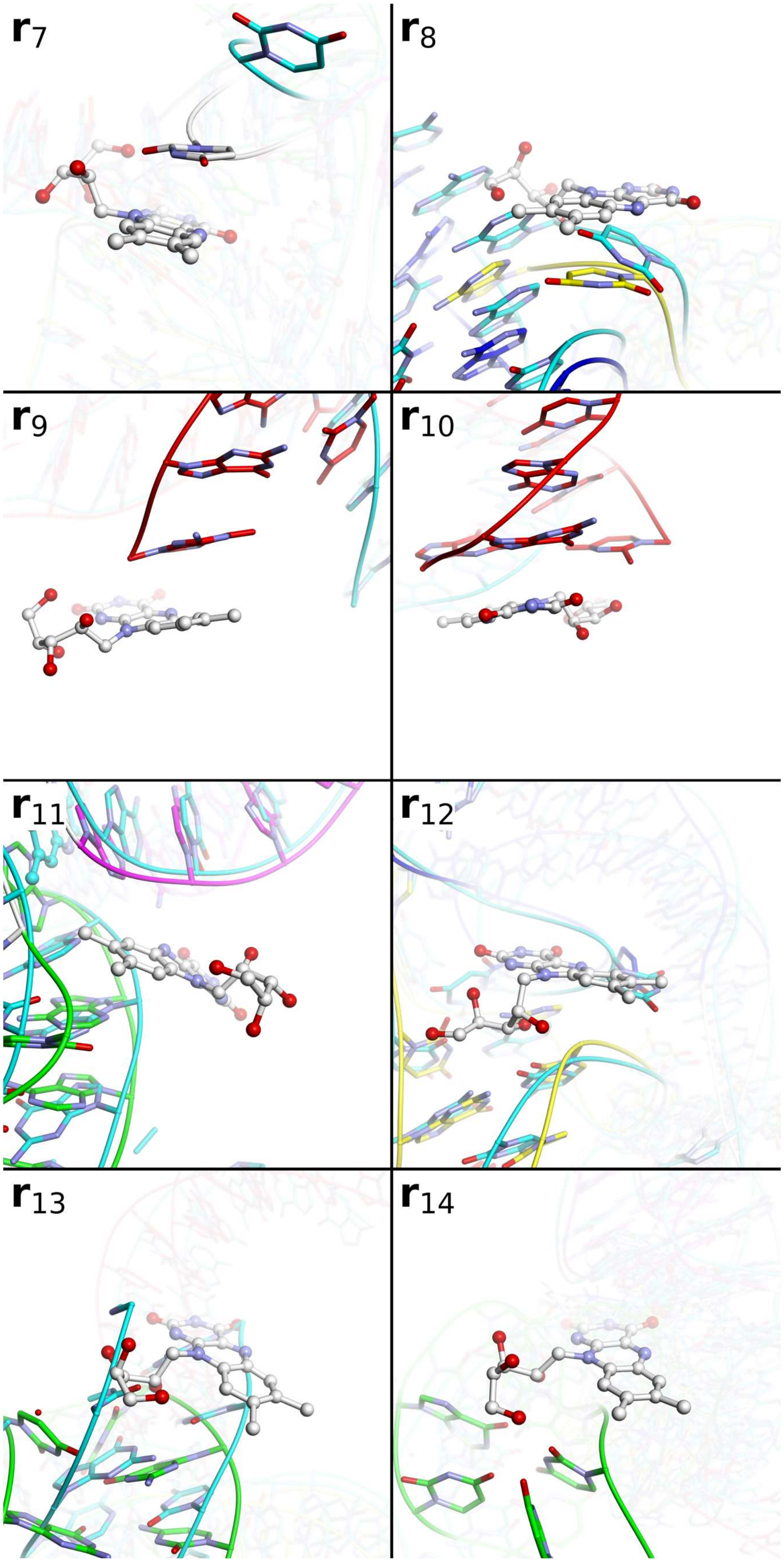

Fig. S3. 3D structure of picked representative configurations $\boldsymbol{r_k}$ and the experimental structure. Representative structures from the dynamic docking simulations $\mathbf{r}_k$ (colored by subdomains, white for the rest) and the experimental structure (cyan, PDB ID 3F4G) are shown with the sidechains of the nearby residues as their front view.

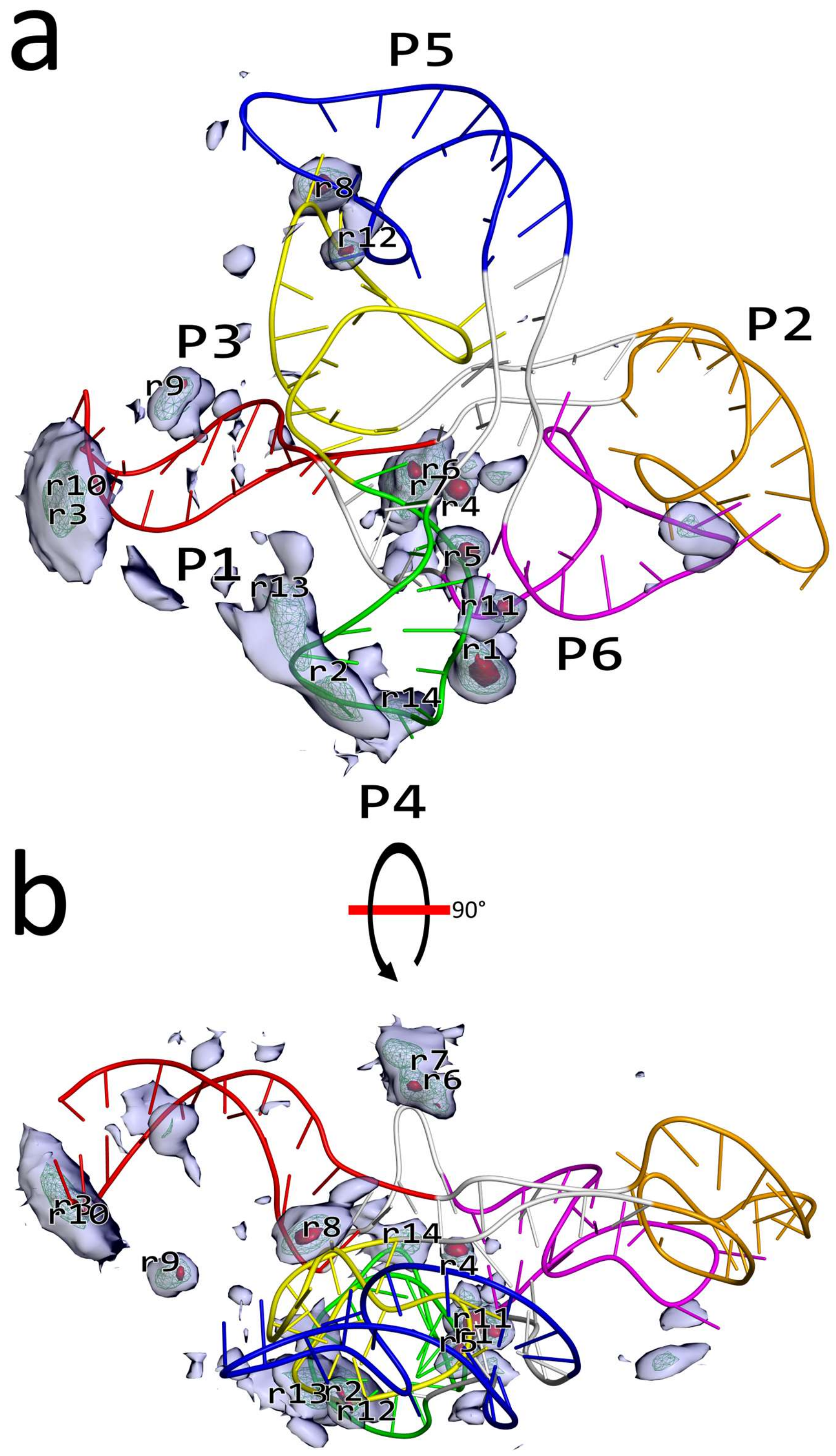

Fig. S4. Density distribution of RBF in 3D around the RNA. A 100 x 100 x 100 grid (each cell has a dimension of 0.963776 x 0.841263 x 0.60446 Å) was created, in which the presence of the ligand (via its center of mass) was measured for the reweighted multicanonical ensemble (300 K). The density was min-max normalized, with the isosurfaces at densities 0.05, 0.01 and 0.001 shown as a red surface, green mesh and blue semi-transparent surface. Also shown is the apo conformation of the RNA along with the aptamer sub-domains colored in red, orange, yellow, green, blue and magenta for P1-P6, respectively. Finally, the center of mass of the ligand in each configuration is labeled by the corresponding $\mathbf{r}_k$. (a) front view like Fig. 1 (b) top view with the molecule rotated 90° about the x-axis.

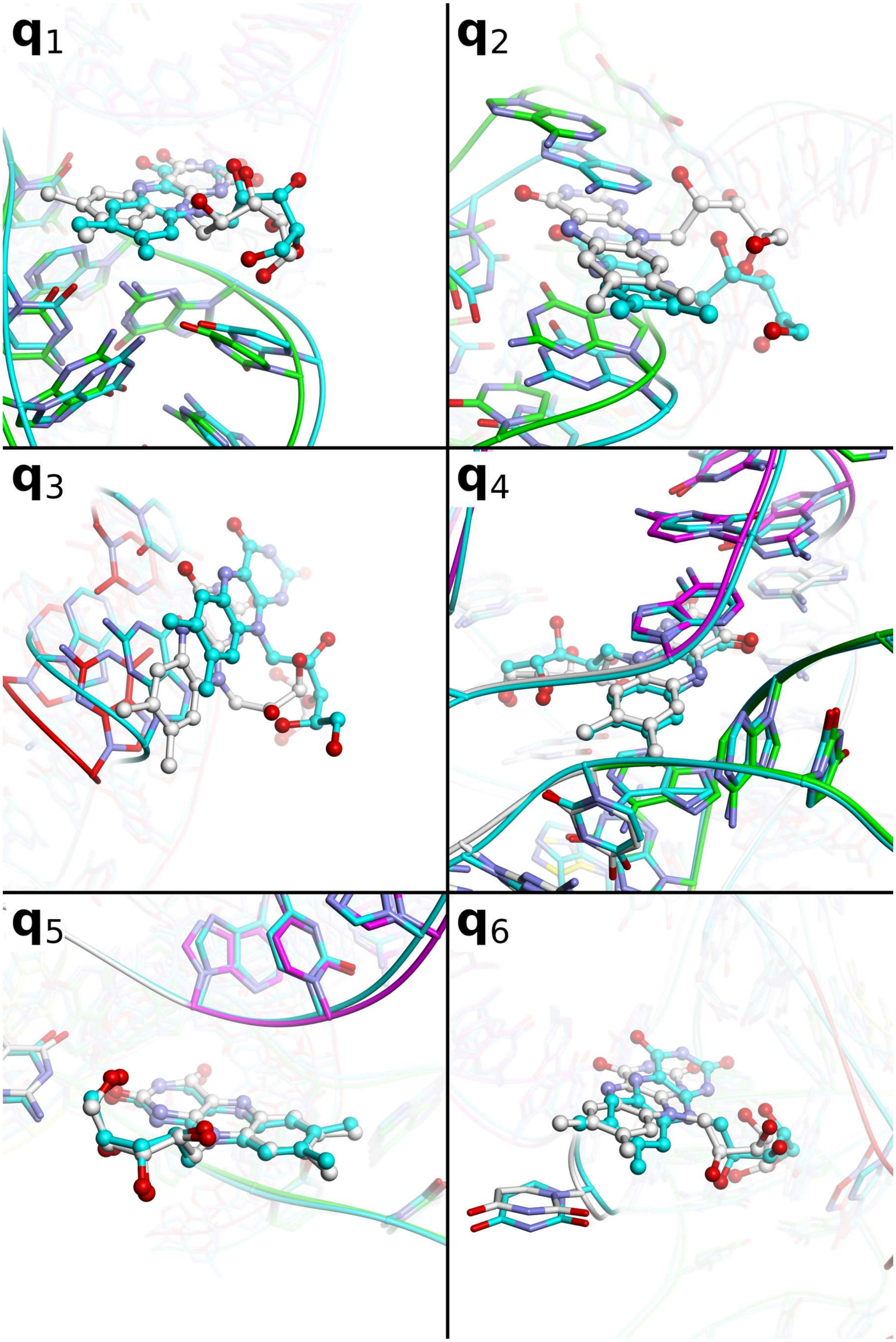
q1
q2
q3
q4
q5
q6

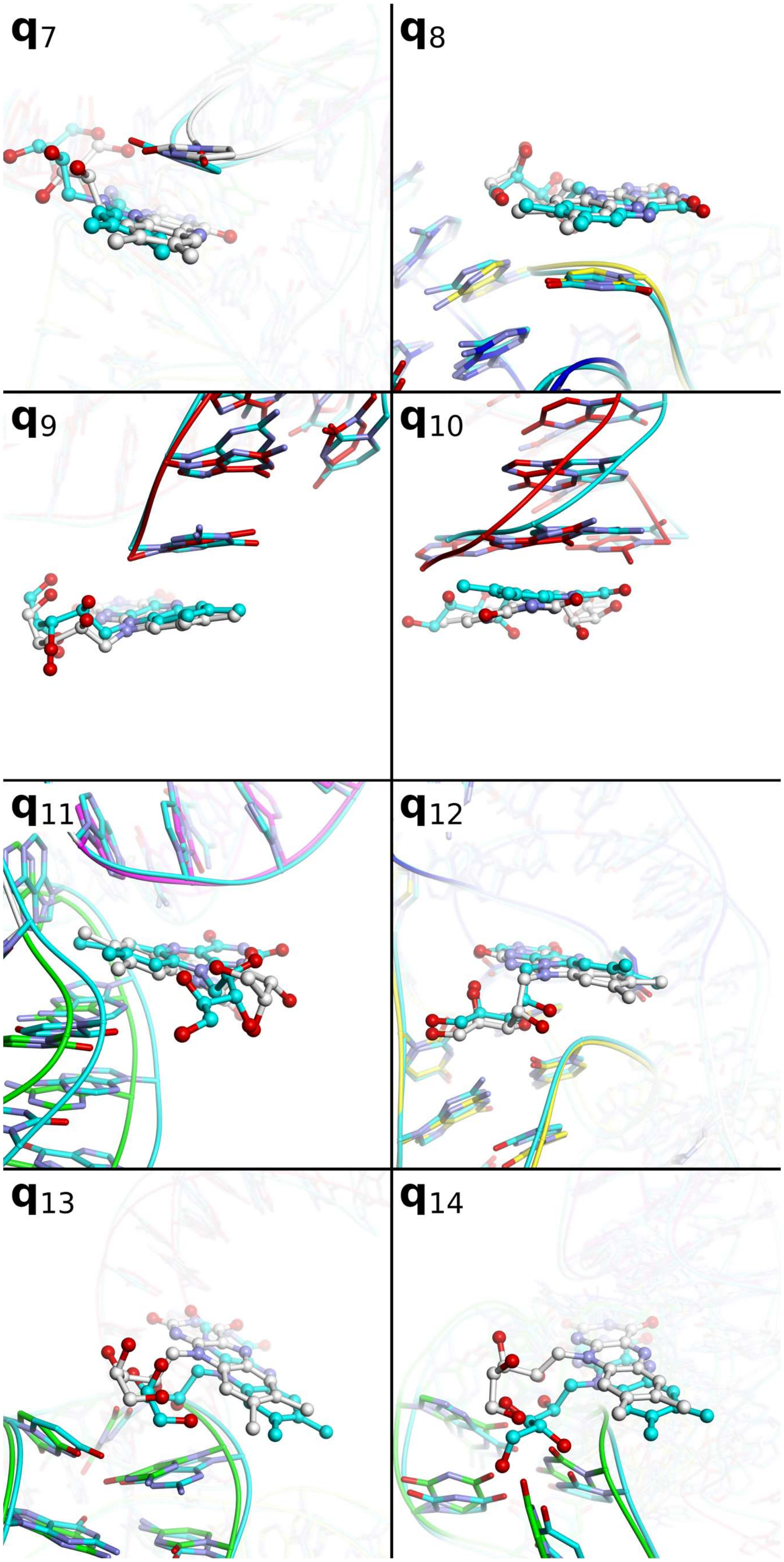

Fig. S5. 3D structure of picked representative configurations $\boldsymbol{r}_k$ and $\boldsymbol{q}_k$. Representative structures from the dynamic docking simulations $\mathbf{r}_k$ (colored by subdomains, white for the rest) and equilibrated structures $\mathbf{q}_k$ (cyan) are shown with the sidechains of the nearby residues are shown as their front view.

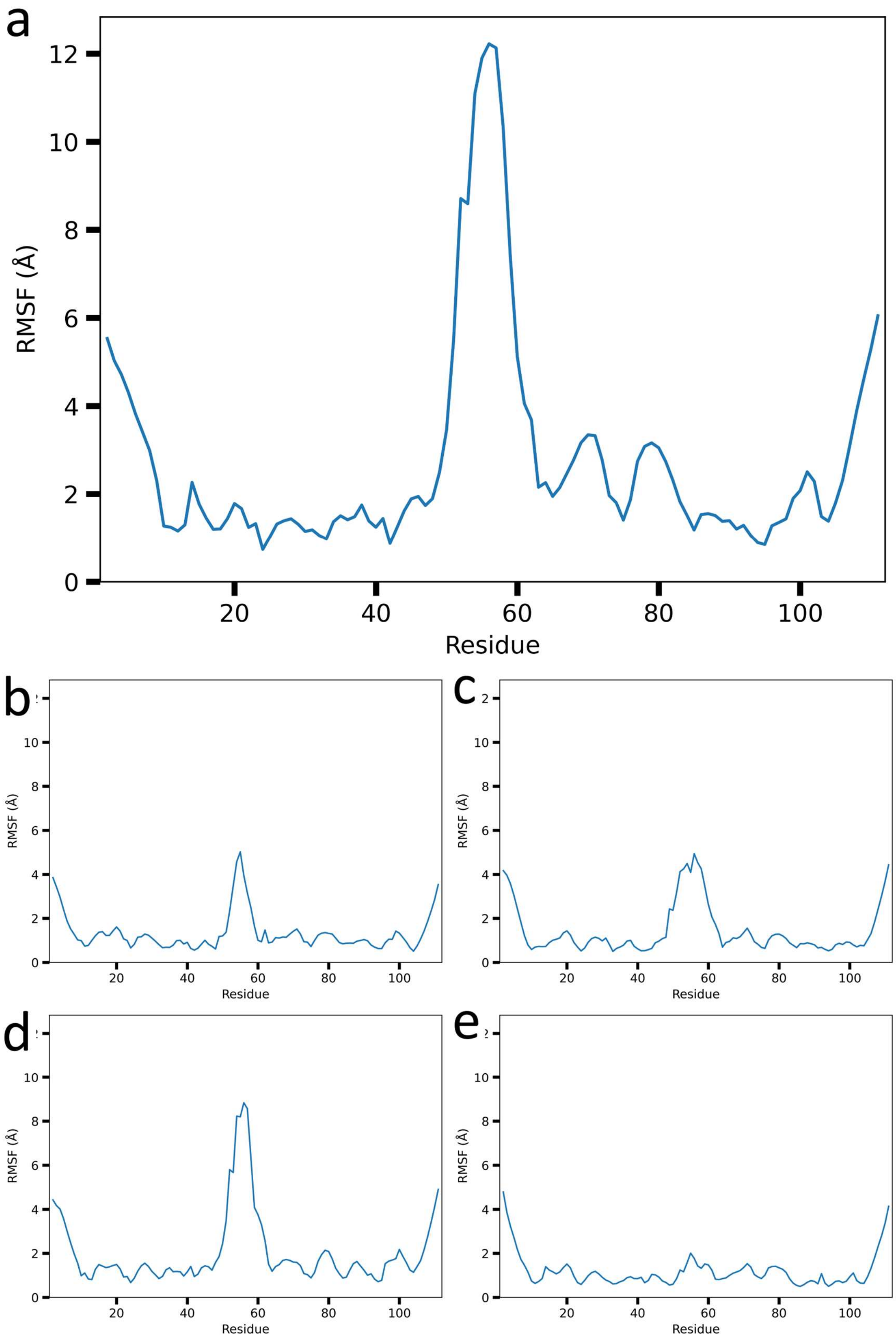

Fig. S6. RMSF analysis of the RNA. Analysis was performed on the reweighted structural ensemble (300K), using all structures (a), $\mathbf{r}_1$ (b), $\mathbf{r}_2$ (c), $\mathbf{r}_3$ (d), $\mathbf{r}_4$ (e).

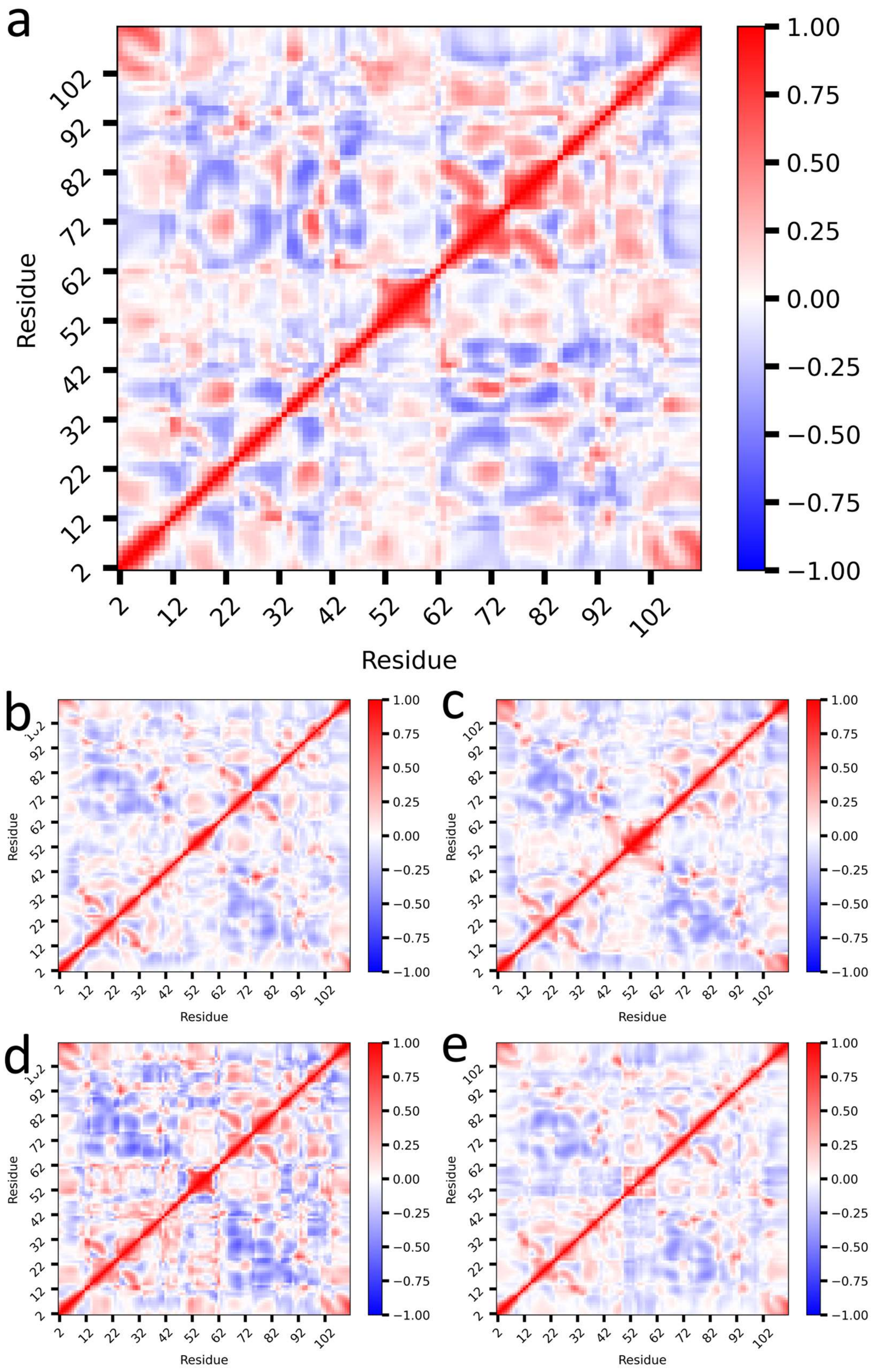

Fig. S7. Dynamic Cross Correlation analysis of the RNA. Analysis was performed on the reweighted structural ensemble (300K), using all structures (a), $\mathbf{r}_1$ (b), $\mathbf{r}_2$ (c), $\mathbf{r}_3$ (d), $\mathbf{r}_4$ (e).

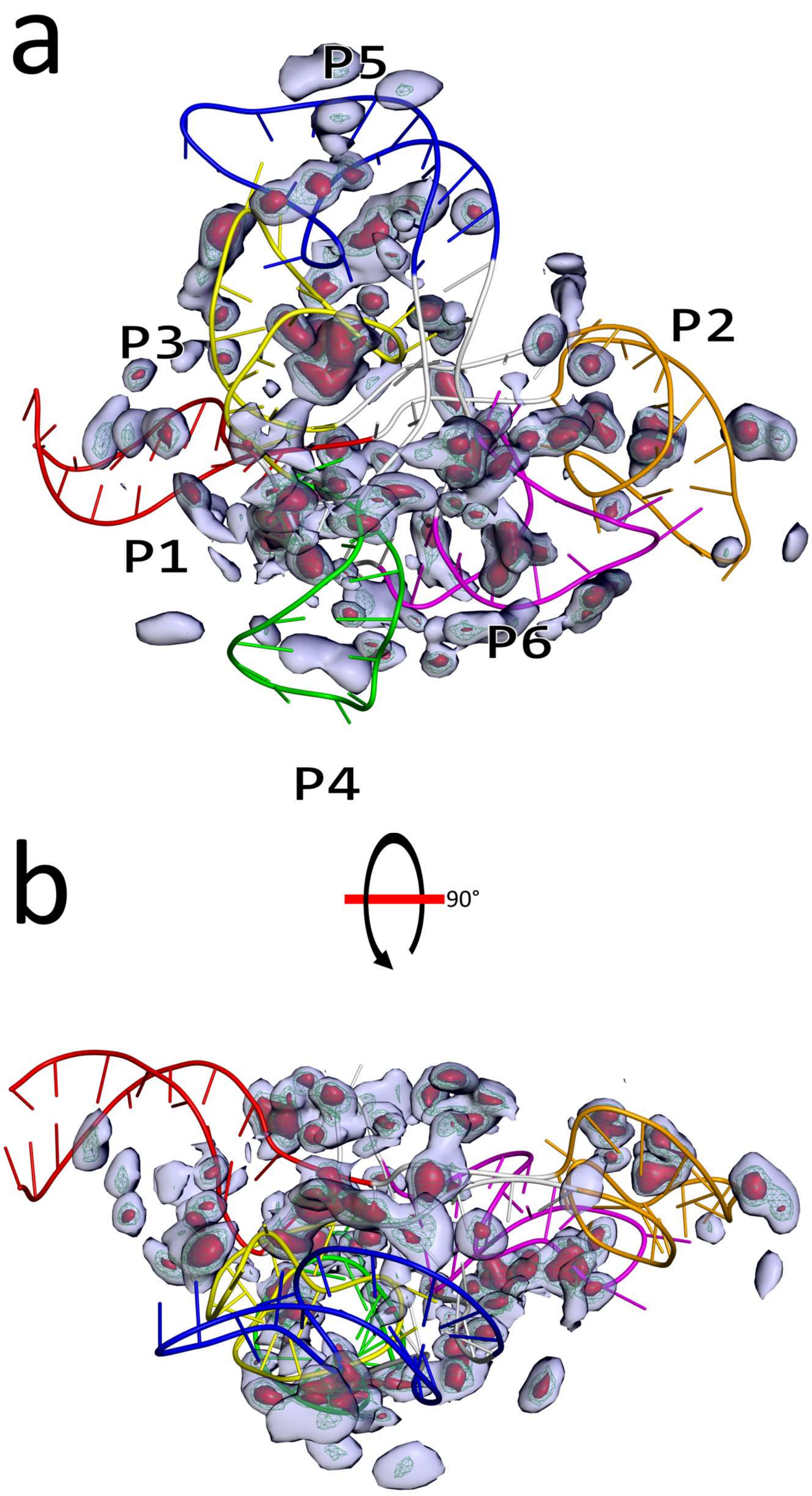
a
P5
P3
P2
P1
P6
P4
b
90°

Fig. S8. Density distribution of Mg^{2+} in 3D around the RNA. A 100 x 100 x 100 grid (each cell has a dimension of 0.963776 x 0.841263 x 0.60446 Å) was created, in which the presence of Mg^{2+} was measured for the reweighted multicanonical ensemble (300 K). The density was min-max normalized, with the isosurfaces at densities 0.1, 0.05 and 0.01 shown as a red surface, green mesh and blue semi-transparent surface. Also shown is the apo conformation of the RNA along with the aptamer sub-domains colored in red, orange, yellow, green, blue and magenta for P1-P6, respectively. (a) front view like Fig. 1 (b) top view with the molecule rotated 90° about the x-axis.

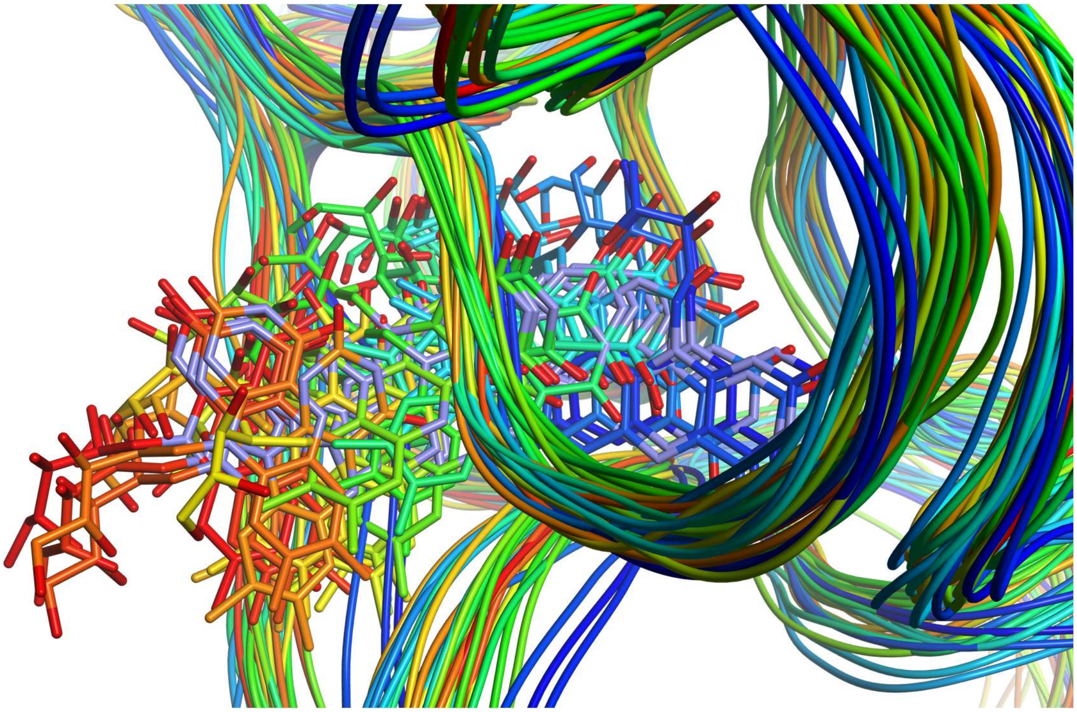

Fig. S9. Overview of binding pathway structures obtained from the multicanonical ensemble. Top view of 19 picked configurations along the binding/unbinding pathway, with the riboswitch and riboflavin colored by their λ-value from the bound state (λ = 0 Å, blue) to the outside state (λ = 14 Å, red) in a blue-red gradient.

Table S1. Convergence of McMD dynamic docking pre-run simulations. [a]

Iteration	Multicanonical coverage (%)	Standard deviation	Simulation length (ns)
0	5.90	4.073	2.00
1	15.10	3.094	0.15
2	28.50	2.304	0.38
3	41.80	1.785	0.71
4	55.60	1.672	1.05
5	69.70	1.432	1.39
6	82.90	1.139	1.74
7	96.50	1.413	2.07
8	100.00	1.377	2.41
9	100.00	0.591	2.50
10	100.00	0.599	12.29
11	100.00	0.944	11.91
12	100.00	0.810	2.68
13	100.00	0.764	4.62
14	100.00	0.674	5.77
15	100.00	0.828	8.72
16	100.00	0.457	4.24
17	100.00	0.306	19.76
18	100.00	0.216	30.73
19	100.00	0.342	38.53
20	100.00	0.352	27.87
21	100.00	0.315	27.09
22	100.00	0.222	30.00
23	100.00	0.238	37.96
24	100.00	0.247	36.49
25	100.00	0.226	35.76
26	100.00	0.159	37.57
27	100.00	0.212	40.00
28	100.00	0.176	38.86
29	100.00	0.159	40.00
30	100.00	0.167	80.00
31	100.00	0.163	80.00
32	100.00	0.173	80.00
33	100.00	0.230	80.00
34	100.00	0.204	80.00

[a] Overview of McMD pre-run, with for each iteration, the multicanonical coverage (i.e., temperature coverage) in percentage (with respect to the full 280 K – 700 K range), flatness of the multicanonical potential energy distribution measured as the standard deviation of the log-probability values corresponding to the energies within the multicanonical range and the simulation length per trajectory (N=30). After achieving a standard deviation of less than 0.2 with a simulation length of 40 ns (iteration #29), five more equilibration iterations at 80 ns are performed to finalize the pre-run.

Table S2. McMD-based dynamic docking results using subsets of the simulation data.[a]

25 % (15 μs)				
	**CFE (kcal/mol)**	**RASA**	**R(exp)-value**	**RMSD (Å)**
$\mathbf{r}_1$	0.00	0.47	0.00	17.99
$\mathbf{r}_2$	0.09	0.63	0.00	35.66
$\mathbf{r}_3$	0.16	0.40	0.00	23.59
$\mathbf{r}_4$	0.17	0.40	0.17	10.67
$\mathbf{r}_5$	0.29	0.59	0.00	22.02
$\mathbf{r}_6$	0.71	0.67	0.00	22.77
$\mathbf{r}_7$	0.80	0.33	0.15	12.32
$\mathbf{r}_8$	0.88	0.58	0.00	35.36
Exp	-	0.16	1.00	0.00

50 % (30 μs)				
	**CFE (kcal/mol)**	**RASA**	**R(exp)-value**	**RMSD (Å)**
$\mathbf{r}_1$	0.00	0.38	0.00	23.66
$\mathbf{r}_2$	0.02	0.46	0.00	17.94
$\mathbf{r}_3$	0.03	0.71	0.00	35.95
$\mathbf{r}_4$	0.27	0.38	0.17	10.68
$\mathbf{r}_5$	0.32	0.59	0.00	22.02
$\mathbf{r}_6$	0.45	0.62	0.00	27.35
$\mathbf{r}_7$	0.52	0.13	1.00	1.23
$\mathbf{r}_8$	0.66	0.67	0.00	21.74
$\mathbf{r}_9$	0.87	0.46	0.01	18.05
$\mathbf{r}_{10}$	0.97	0.72	0.00	23.23
$\mathbf{r}_{11}$	0.98	0.76	0.00	23.57
Exp	-	0.16	1.00	0.00

75 % (45 μs)				
	**CFE (kcal/mol)**	**RASA**	**R(exp)-value**	**RMSD (Å)**
$\mathbf{r}_1$	0.00	0.46	0.00	17.67
$\mathbf{r}_2$	0.04	0.42	0.00	23.04
$\mathbf{r}_3$	0.07	0.59	0.00	19.78
$\mathbf{r}_4$	0.17	0.71	0.00	35.95
$\mathbf{r}_5$	0.25	0.14	1.00	2.28
$\mathbf{r}_6$	0.31	0.36	0.21	10.10
$\mathbf{r}_7$	0.48	0.64	0.00	27.21
$\mathbf{r}_8$	0.57	0.62	0.00	31.35
$\mathbf{r}_9$	0.73	0.66	0.00	21.79
$\mathbf{r}_{10}$	0.93	0.65	0.00	37.39
Exp	-	0.16	1.00	0.00

[a] The McMD-based dynamic docking ensemble was re-analyzed using subsets of the simulation data, starting from the PCA to the analysis of the top ranked (less than 1.0 kcal/mol) structures $\mathbf{r}_k$, with the statistics of those structures shown in the table. E.g., for the 25 % (15 μs) dataset, 500 ns of each trajectory (30 parallel trajectories) was used for the PCA, after which those snapshots were re-clustered using those PC coordinates, to finally obtain the representative structures.

Table S3. RMSDs of riboswitch's residues during binding of the ligand RBF along λ.[α]

λ (Å)	G11	A48	A49	U61	G62	G84	A85	G93	G98	A99	G101
**-0.5**	0.29	0.85	0.7	0.43	0.6	0.7	0.37	0.53	0.94	0.88	0.92
	(1.52)	(2.48)	(2.33)	(3.96)	(3.83)	(1.47)	(1.43)	(0.99)	(1.19)	(1.0)	(1.48)
**0.0**	0.0	0.0	0.0	0.0	0.0	0.0	0.0	0.0	0.0	0.0	0.0
	(1.6)	(2.18)	(1.84)	(3.82)	(4.0)	(1.74)	(1.42)	(1.07)	(1.11)	(1.11)	(1.78)
**0.5**	0.87	0.85	0.67	1.41	0.78	0.61	0.46	0.61	0.63	0.48	1.16
	(1.42)	(2.2)	(2.06)	(2.91)	(4.25)	(1.98)	(1.63)	(1.28)	(1.52)	(1.27)	(2.02)
**1.0**	0.39	1.8	2.73	4.87	1.62	1.0	0.85	0.94	1.96	1.92	2.24
	(1.52)	(2.9)	(3.8)	(4.66)	(4.64)	(1.42)	(1.41)	(0.76)	(1.96)	(1.86)	(2.63)
**1.5**	1.21	1.9	3.12	5.29	1.8	2.07	2.13	1.41	2.47	2.77	1.9
	(1.14)	(2.73)	(3.95)	(5.2)	(3.51)	(0.74)	(1.93)	(0.55)	(2.57)	(2.8)	(2.64)
**2.0**	1.36	2.01	3.22	5.88	2.94	1.71	2.15	1.82	1.77	2.23	1.37
	(0.8)	(3.32)	(4.16)	(5.22)	(5.11)	(0.94)	(1.67)	(1.59)	(2.25)	(2.74)	(2.38)
**2.5**	1.0	2.26	3.14	6.25	3.23	1.38	1.85	1.27	1.07	1.32	1.35
	(0.77)	(3.31)	(4.07)	(5.84)	(5.39)	(0.93)	(1.45)	(0.99)	(1.68)	(1.91)	(2.21)
**3.0**	1.57	2.79	2.87	6.44	4.26	2.2	2.38	1.79	2.26	2.8	1.45
	(1.23)	(3.57)	(3.57)	(5.69)	(4.97)	(0.98)	(1.82)	(0.84)	(2.36)	(2.75)	(2.43)
**3.5**	0.96	2.5	3.14	6.25	3.37	1.5	2.15	0.85	1.9	2.1	1.45
	(1.56)	(3.7)	(4.21)	(6.01)	(5.39)	(0.88)	(1.83)	(0.87)	(2.35)	(2.2)	(1.96)
**4.0**	1.22	2.11	2.88	4.97	2.98	2.03	2.2	1.14	1.5	1.34	1.23
	(1.06)	(3.16)	(3.8)	(4.31)	(5.59)	(1.09)	(1.95)	(0.81)	(1.59)	(1.43)	(1.65)
**4.5**	1.61	2.27	3.02	5.63	3.18	1.89	2.51	1.76	1.83	1.59	1.05
	(1.24)	(3.43)	(3.92)	(4.3)	(5.53)	(0.85)	(1.99)	(1.12)	(2.07)	(1.9)	(1.94)
**5.0**	2.18	2.23	3.2	6.2	3.58	2.12	2.41	1.64	2.08	1.98	1.2
	(1.95)	(3.59)	(4.17)	(4.92)	(5.93)	(0.99)	(1.89)	(1.0)	(2.07)	(2.08)	(2.32)
**5.5**	1.91	1.54	2.51	6.22	3.79	3.1	1.91	2.0	1.89	2.51	1.83
	(1.47)	(2.91)	(3.48)	(4.99)	(5.93)	(1.93)	(0.93)	(1.17)	(1.33)	(2.03)	(2.12)
**6.0**	2.97	1.94	2.7	7.64	4.44	3.77	2.6	2.28	2.11	2.29	1.73
	(2.2)	(3.09)	(3.49)	(6.24)	(6.43)	(2.41)	(1.52)	(1.48)	(1.74)	(2.04)	(2.67)
**6.5**	2.47	2.04	2.81	7.64	4.91	3.99	2.63	2.59	2.36	2.64	1.72
	(1.76)	(3.24)	(3.63)	(6.72)	(6.77)	(2.65)	(1.65)	(1.7)	(1.94)	(2.32)	(2.42)
**7.0**	1.84	1.42	2.79	4.77	2.41	3.77	2.08	2.06	2.08	2.5	2.36
	(1.32)	(2.33)	(3.46)	(3.19)	(5.16)	(2.29)	(0.95)	(1.18)	(1.8)	(2.13)	(2.75)
**7.5**	2.49	1.99	2.93	7.21	4.4	4.34	2.77	2.69	2.77	3.25	1.75
	(1.83)	(3.09)	(3.51)	(6.02)	(6.19)	(3.03)	(1.8)	(1.85)	(2.46)	(2.85)	(2.38)
**8.0**	2.58	1.78	2.52	5.84	3.61	2.89	2.08	1.58	2.75	2.72	2.3
	(1.8)	(2.61)	(3.12)	(4.96)	(5.77)	(1.84)	(1.06)	(0.9)	(2.09)	(2.21)	(2.42)
**8.5**	1.45	2.01	2.83	5.14	4.01	2.6	2.26	1.7	1.94	2.84	1.73
	(1.56)	(3.37)	(3.75)	(4.16)	(6.79)	(1.66)	(1.52)	(1.38)	(1.66)	(2.59)	(2.49)
**9.0**	1.52	1.75	2.76	5.92	4.18	2.75	2.32	2.01	1.65	1.98	1.94
	(1.34)	(2.76)	(3.62)	(5.07)	(6.9)	(1.79)	(1.69)	(1.3)	(1.47)	(1.59)	(2.15)
**9.5**	1.82	2.1	2.74	5.6	3.97	2.91	2.33	1.97	1.87	2.42	2.09
	(1.29)	(2.56)	(3.33)	(4.87)	(6.48)	(1.83)	(1.41)	(1.02)	(1.7)	(2.1)	(2.36)
**10.0**	2.3	2.66	2.67	6.37	3.93	2.82	2.44	2.1	1.88	2.19	1.54
	(1.41)	(3.14)	(3.21)	(5.16)	(5.9)	(1.78)	(1.77)	(1.2)	(2.28)	(2.35)	(2.63)
**10.5**	1.79	1.44	3.16	5.2	2.64	3.07	2.31	2.08	2.15	2.45	1.86
	(1.28)	(2.48)	(3.97)	(3.89)	(4.83)	(1.71)	(2.2)	(1.15)	(2.61)	(2.88)	(2.61)
**11.0**	2.26	1.82	3.09	6.35	3.96	3.87	3.1	2.38	2.63	3.1	1.5
	(1.2)	(2.64)	(3.77)	(4.93)	(5.87)	(2.55)	(2.51)	(1.43)	(2.8)	(3.23)	(2.84)
**11.5**	2.36	1.66	3.16	4.71	4.26	3.1	2.5	2.04	1.61	1.75	1.24
	(1.83)	(2.62)	(4.02)	(4.67)	(4.37)	(1.8)	(2.02)	(1.12)	(1.95)	(2.26)	(2.39)
**12.0**	2.76	1.97	2.37	6.41	3.95	2.73	2.4	1.93	1.89	2.11	1.74
	(1.85)	(2.36)	(2.81)	(4.74)	(5.49)	(1.35)	(1.9)	(1.06)	(2.45)	(2.62)	(2.97)

**12.5**	1.54	1.92	2.39	4.66	2.58	1.55	1.75	1.18	2.01	2.13	2.47
	(1.11)	(2.54)	(3.32)	(2.99)	(3.92)	(1.31)	(1.1)	(0.62)	(1.81)	(2.09)	(2.61)
**13.0**	1.64	1.86	2.75	5.1	3.25	1.98	2.79	1.91	2.13	2.5	2.03
	(1.13)	(2.37)	(3.37)	(4.05)	(4.56)	(1.15)	(1.92)	(1.03)	(1.83)	(2.19)	(2.48)
**13.5**	1.58	2.25	2.83	6.02	3.26	1.56	2.43	1.55	2.34	2.78	2.0
	(0.68)	(3.08)	(3.58)	(4.08)	(4.78)	(0.86)	(1.67)	(0.65)	(2.09)	(2.58)	(2.62)
**14.0**	1.15	1.95	2.5	5.69	3.16	1.59	2.21	1.42	2.0	2.34	2.18
	(0.88)	(2.73)	(3.24)	(3.58)	(4.56)	(1.26)	(1.27)	(0.5)	(1.83)	(2.07)	(2.67)

[α] Shown are the RMSDs in Å with respect to $\mathbf{r}_4$ (and the experimental structure in parentheses) for the nucleotides in and around the pocket for the structures shown in Fig. S8.

Table S4. System & simulation parameters for Riboswitch – RBF binding simulations.

Parameter	Value
**Number of atoms (total)**	49341
**Number of atoms (RNA)**	3572
**Number of atoms (RBF)**	48
**Number of water molecules**	15191
**Number of K^+**	106
**Number of Mg^{2+}**	15
**Salt concentration**	0.1 M KCl
**Box size (pre-NPT)**	97.4444 x 85.0575 x 61.1150 Å
**Box size (post-NPT)**	96.3776 x 84.1263 x 6.04460 Å
**Thermostat**	Bussi (V-rescale)
**Barostat (NPT only)**	Bussi (C-rescale)
**Electrostatics**	Zero-Dipole
**Cutoff (LJ & elec)**	12 Å
**Timestep**	2 fs
**Constraints**	LINCS for solute, SETTLE for waters
**McMD parallel trajectories**	30
**McMD temperature range**	280 K – 700 K
**Pre-run simulation time**	0.90525 µs per trajectory
**Production-run simulation time**	2.0 µs per trajectory

SUPPLEMENTARY REFERENCES

(1) Nakajima, N.; Nakamura, H.; Kidera, A. Multicanonical Ensemble Generated by Molecular Dynamics Simulation for Enhanced Conformational Sampling of Peptides. *J. Phys. Chem. B* **1997**, *101* (5), 817–824. https://doi.org/10.1021/jp962142e.

(2) Nakajima, N.; Higo, J.; Kidera, A.; Nakamura, H. Free Energy Landscapes of Peptides by Enhanced Conformational Sampling 1 1Edited by B. Honig. *J. Mol. Biol.* **2000**, *296* (1), 197–216. https://doi.org/10.1006/jmbi.1999.3440.

(3) Higo, J.; Ito, N.; Kuroda, M.; Ono, S.; Nakajima, N.; Nakamura, H. Energy Landscape of a Peptide Consisting of α-Helix, 3 $_{10}$ -Helix, β-Turn, β-Hairpin, and Other Disordered Conformations. *Protein Sci.* **2001**, *10* (6), 1160–1171. https://doi.org/10.1110/ps.44901.

(4) Kamiya, N.; Higo, J.; Nakamura, H. Conformational Transition States of a β-Hairpin Peptide between the Ordered and Disordered Conformations in Explicit Water. *Protein Sci.* **2002**, *11* (10), 2297–2307. https://doi.org/10.1110/ps.0213102.

(5) Ikeda, K.; Higo, J. Free-Energy Landscape of a Chameleon Sequence in Explicit Water and Its Inherent α/β Bifacial Property. *Protein Sci.* **2009**, *12* (11), 2542–2548. https://doi.org/10.1110/ps.03143803.

(6) Kamiya, N.; Yonezawa, Y.; Nakamura, H.; Higo, J. Protein-Inhibitor Flexible Docking by a Multicanonical Sampling: Native Complex Structure with the Lowest Free Energy and a Free-Energy Barrier Distinguishing the Native Complex from the Others. *Proteins* **2008**, *70* (1), 41–53. https://doi.org/10.1002/prot.21409.

(7) Ikebe, J.; Umezawa, K.; Kamiya, N.; Sugihara, T.; Yonezawa, Y.; Takano, Y.; Nakamura, H.; Higo, J. Theory for Trivial Trajectory Parallelization of Multicanonical Molecular Dynamics and Application to a Polypeptide in Water. *J. Comput. Chem.* **2011**, *32* (7), 1286–1297. https://doi.org/10.1002/jcc.21710.

(8) Bekker, G.-J.; Kamiya, N.; Araki, M.; Fukuda, I.; Okuno, Y.; Nakamura, H. Accurate Prediction of Complex Structure and Affinity for a Flexible Protein Receptor and Its Inhibitor. *J. Chem. Theory Comput.* **2017**, *13* (6), 2389–2399. https://doi.org/10.1021/acs.jctc.6b01127.

(9) Bekker, G.-J.; Kamiya, N. Dynamic Docking Using Multicanonical Molecular Dynamics: Simulating Complex Formation at the Atomistic Level. In *Protein-Ligand Interactions and Drug Design*; Ballante, F., Ed.; Methods in Molecular Biology; Springer US: New York, NY, 2021; Vol. 2266, pp 187–202. https://doi.org/10.1007/978-1-0716-1209-5_11.

(10) Bekker, G.-J.; Kamiya, N. Advancing the Field of Computational Drug Design Using Multicanonical Molecular Dynamics-Based Dynamic Docking. *Biophys. Rev.* **2022**, *14* (6), 1349–1358. https://doi.org/10.1007/s12551-022-01010-z.

(11) Numoto, N.; Kamiya, N.; Bekker, G.-J.; Yamagami, Y.; Inaba, S.; Ishii, K.; Uchiyama, S.; Kawai, F.; Ito, N.; Oda, M. Structural Dynamics of the PET-Degrading Cutinase-like Enzyme from *Saccharomonospora Viridis* AHK190 in Substrate-Bound States Elucidates the Ca^{2+}-Driven Catalytic Cycle. *Biochemistry* **2018**, *57* (36), 5289–5300. https://doi.org/10.1021/acs.biochem.8b00624.

(12) Bekker, G.-J.; Araki, M.; Oshima, K.; Okuno, Y.; Kamiya, N. Dynamic Docking of a Medium-Sized Molecule to Its Receptor by Multicanonical MD Simulations. *J. Phys. Chem. B* **2019**, *123* (11), 2479–2490. https://doi.org/10.1021/acs.jpcb.8b12419.

(13) Bekker, G.-J.; Araki, M.; Oshima, K.; Okuno, Y.; Kamiya, N. Exhaustive Search of the Configurational Space of Heat-shock Protein 90 with Its Inhibitor by Multicanonical

Molecular Dynamics Based Dynamic Docking. *J. Comput. Chem.* **2020**, *41* (17), 1606–1615. https://doi.org/10.1002/jcc.26203.
(14) Best, R. B.; Hummer, G.; Eaton, W. A. Native Contacts Determine Protein Folding Mechanisms in Atomistic Simulations. *Proc. Natl. Acad. Sci.* **2013**, *110* (44), 17874–17879. https://doi.org/10.1073/pnas.1311599110.
(15) Bekker, G.-J.; Ma, B.; Kamiya, N. Thermal Stability of Single-Domain Antibodies Estimated by Molecular Dynamics Simulations. *Protein Sci.* **2019**, *28* (2), 429–438. https://doi.org/10.1002/pro.3546.
(16) Bekker, G.-J.; Fukuda, I.; Higo, J.; Fukunishi, Y.; Kamiya, N. Cryptic-Site Binding Mechanism of Medium-Sized Bcl-xL Inhibiting Compounds Elucidated by McMD-Based Dynamic Docking Simulations. *Sci. Rep.* **2021**, *11* (1), 5046. https://doi.org/10.1038/s41598-021-84488-z.
